# A secondary analysis of the SODa-BIC randomized clinical trial using multiple hierarchical composite outcomes

**DOI:** 10.62675/2965-2774.20260279

**Published:** 2026-09-17

**Authors:** Humphrey G. M. Walker, Alastair Brown, Kyle C. White, Mairead McNamara, D. Jamie Cooper, Tomoko Fujii, Alisa M. Higgins, Carol L. Hodgson, Leanlove Navarra, Alistair Nichol, Sandra Peake, Alvaro Rea-Neto, Paul Secombe, Fiona Osborne, Hugh Burrill, Emily See, Meredith Young, Fernando Godinho Zampieri, Mark Plummer, Connor Christie, Leo Nunnink, Josephine Mackay, Shay McGuinness, Joradee Marmol, Ravindranath Tiruvoipati, Michael Bailey, Paul J. Young, Andrew Udy, Ary Serpa

**Affiliations:** 1 St Vincent's Hospital Department of Critical Care Melbourne VIC Australia Department of Critical Care, St Vincent's Hospital - Melbourne, VIC, Australia.; 2 Epworth Hospital Richmond Intensive Care Unit Melbourne VIC Australia Intensive Care Unit, Epworth Hospital Richmond - Melbourne, VIC, Australia.; 3 University of Melbourne Department of Critical Care Melbourne VIC Australia Department of Critical Care, University of Melbourne - Melbourne, VIC, Australia.; 4 Monash University School of Public Health and Preventive Medicine Australian and New Zealand Intensive Care Research Centre Melbourne VIC Australia Australian and New Zealand Intensive Care Research Centre, School of Public Health and Preventive Medicine, Monash University - Melbourne, VIC, Australia.; 5 Queensland University of Technology Faculty of Health School of Clinical Sciences Brisbane QLD Australia School of Clinical Sciences, Faculty of Health, Queensland University of Technology - Brisbane, QLD, Australia.; 6 Princess Alexandra Hospital Woolloongabba QLD Australia Intensive Care Unit, Princess Alexandra Hospital - Woolloongabba, QLD, Australia.; 7 The Alfred Hospital Department of Intensive Care and Hyperbaric Medicine Melbourne VIC Australia Department of Intensive Care and Hyperbaric Medicine, The Alfred Hospital - Melbourne, VIC, Australia.; 8 Jikei University Hospital Department of Intensive Care Tokyo Japan Department of Intensive Care, Jikei University Hospital - Tokyo, Japan.; 9 Wellington Hospital Intensive Care Unit Wellington New Zealand Intensive Care Unit, Wellington Hospital - Wellington, New Zealand.; 10 Medical Research Institute of New Zealand Wellington New Zealand Medical Research Institute of New Zealand - Wellington, New Zealand.; 11 University College Dublin Clinical Research Centre at St. Vincent's University Hospital Dublin Ireland Clinical Research Centre, University College Dublin Clinical Research Centre at St. Vincent's University Hospital - Dublin, Ireland.; 12 The Queen Elizabeth Hospital Department of Intensive Care Medicine Woodville South SA Australia Department of Intensive Care Medicine, The Queen Elizabeth Hospital - Woodville South, SA, Australia.; 13 University of Adelaide Faculty of Health and Medical Sciences Adelaide SA Australia Faculty of Health and Medical Sciences, University of Adelaide - Adelaide, SA, Australia.; 14 Universidade Federal do Paraná Department of Clinical Medicine Curitiba PR Brazil Department of Clinical Medicine, Universidade Federal do Paraná - Curitiba (PR), Brazil.; 15 Alice Springs Hospital Intensive Care Unit Alice Springs NT Australia Intensive Care Unit, Alice Springs Hospital - Alice Springs, NT, Australia.; 16 Australian and New Zealand Intensive Care Society Centre for Outcome and Resource Evaluation Prahran VIC Australia Australian and New Zealand Intensive Care Society Centre for Outcome and Resource Evaluation - Prahran, VIC, Australia.; 17 Royal Melbourne Hospital Department of Intensive Care Melbourne VIC Australia Department of Intensive Care, Royal Melbourne Hospital - Melbourne, VIC, Australia.; 18 University of Alberta Faculty of Medicine and Dentistry Department of Critical Care Medicine Edmonton AB Canada Department of Critical Care Medicine, Faculty of Medicine and Dentistry, University of Alberta - Edmonton, AB, Canada.; 19 University of Adelaide Faculty of Health and Medical Sciences Adelaide Medical School Adelaide SA Australia Adelaide Medical School, Faculty of Health and Medical Sciences, University of Adelaide - Adelaide, SA, Australia.; 20 Royal Adelaide Hospital Department of Intensive Care Medicine Adelaide SA Australia Department of Intensive Care Medicine, Royal Adelaide Hospital - Adelaide, SA, Australia.; 21 University of Queensland Faculty of Medicine, Mayne Academy of Critical Care St Lucia QLD Australia Faculty of Medicine, Mayne Academy of Critical Care, University of Queensland - St Lucia, QLD, Australia.; 22 Auckland City Hospital Cardiothoracic and Vascular Intensive Care Unit Auckland New Zealand Cardiothoracic and Vascular Intensive Care Unit, Auckland City Hospital - Auckland, New Zealand.; 23 Frankston Hospital Department of Intensive Care Medicine Frankston VIC Australia Department of Intensive Care Medicine, Frankston Hospital - Frankston, VIC, Australia.; 24 Monash University Peninsula Clinical School Frankston VIC Australia Division of Medicine, Peninsula Clinical School, Monash University - Frankston, VIC, Australia.; 25 Peninsula Private Hospital Department of Intensive Care Medicine Langwarrin VIC Australia Department of Intensive Care Medicine, Peninsula Private Hospital, Ramsay Health - Langwarrin, VIC, Australia.; 26 Austin Hospital Department of Intensive Care Heidelberg VIC Australia Department of Intensive Care, Austin Hospital - Heidelberg, VIC, Australia.; 27 Hospital Israelita Albert Einstein Department of Critical Care Medicine São Paulo SP Brazil Department of Critical Care Medicine, Hospital Israelita Albert Einstein - São Paulo (SP), Brazil.

**Keywords:** Critical Illness, Length of stay, Sodium bicarbonate, Renal replacement therapy, Acute kidney injury, Acidosis, Probability, Hospitals, Kidney, Intensive care units

## Abstract

**Background:**

The Sodium Bicarbonate for Metabolic Acidosis in the ICU (SODa-BIC) trial assessed the effect of sodium bicarbonate on Major Adverse Kidney Events at 30 days (MAKE30) in critically ill patients with metabolic acidosis. Although the trial demonstrated no significant difference in the primary outcome, the individual components showed differing magnitudes and directions of effect; we undertook an exploratory analysis of the SODa-BIC trial using different hierarchical outcomes to better understand potential treatment effects of sodium bicarbonate.

**Methods:**

Post-hoc analysis of the SODa-BIC trial. All patients included in the SODa-BIC trial were eligible. The primary outcome was a win ratio analysis with a hierarchy consisting of death, requirement for renal replacement therapy, persistent renal dysfunction, and hospital length of stay. Several sensitivity and subgroup analyses were conducted.

**Results:**

The primary analysis resulted in 59,250 pairs from 488 patients. Consistent with the SODa-BIC analysis, there was no overall evidence of a treatment effect of sodium bicarbonate, with a win ratio of 0.97 (95%CI 0.79 - 1.19) and a Desirability of Outcome Ranking probability of 0.49 (95%CI 0.45 - 0.54). A majority of pairwise comparisons were resolved by mortality and hospital length of stay. Evidence of significant treatment effect modification was observed across subgroups defined by acute kidney injury and severity of acidemia.

**Conclusion:**

In this post-hoc analysis of the SODa-BIC trial, the use of hierarchical composite outcomes did not alter the neutral overall interpretation of the original trial. However, this analysis provides additional insight into the relative contribution of individual components of the primary outcome.

## INTRODUCTION

The Sodium Bicarbonate for Metabolic Acidosis in the ICU (SODa-BIC trial) evaluated sodium bicarbonate therapy in critically ill patients with metabolic acidosis requiring vasopressor support.^(1)^ The primary outcome was Major Adverse Kidney Events within 30 days (MAKE30), a composite of death, receipt of renal replacement therapy (RRT), and persistent renal dysfunction within 30 days. Although the trial demonstrated no significant difference in the primary outcome, the individual components showed differing magnitudes and directions of effect, consistent with previous studies suggesting possible reductions in kidney-related complications despite uncertain effects on mortality.^(2,3)^

Composite outcomes are frequently used in critical care trials to improve efficiency and increase event rates.^(4,5)^ However, conventional composite outcomes assign equal importance to all components and may obscure clinically meaningful differences between outcomes of substantially different relevance, such as death and persistent renal dysfunction.^(6,7)^ As a result, interpretation of treatment effects may be challenging, particularly when individual components occur with different frequencies or demonstrate heterogeneous treatment effects.^(8)^

Hierarchical composite outcomes have been proposed as an alternative to prioritize clinically important events. A win ratio compares patients according to a predefined hierarchy of outcomes, while the Desirability of Outcome Ranking (DOOR) framework classifies patients according to ordered levels of overall clinical benefit.^(7,9)^ These methods may provide additional insights into treatment effects by preserving the relative importance of individual outcome components and capturing the full spectrum of patient outcomes.

We therefore performed a post hoc analysis of the SODa-BIC trial using both win ratio and DOOR methodologies. Our objectives were to determine whether hierarchical analyses altered the interpretation of the original trial findings and to better understand the contribution of individual kidney-related and mortality outcomes to the overall treatment effect.

## METHODS

### Study design population

This study was a post hoc analysis of the SODa-BIC randomized clinical trial. The design and primary results of the parent trial have been reported previously.^(1)^ Briefly, SODa-BIC was an international, multicenter, double-blind, placebo-controlled trial that enrolled critically ill adults with metabolic acidosis requiring continuous vasopressor support. Eligible patients were ≥ 18 years of age, receiving a continuous vasopressor infusion to maintain a mean arterial pressure above 65mmHg, and had metabolic acidosis defined by a pH < 7.30, base excess ≤ -4mEq/L, and partial pressure of carbon dioxide in arterial blood (PaCO_2_) ≤ 45mmHg if not mechanically ventilated or ≤ 50mmHg if mechanically ventilated. Participants were randomly assigned in a 1:1 ratio to receive either sodium bicarbonate or placebo (5% dextrose) administered over five hours (Figures 1S and 2S - Supplementary Material). The primary endpoint of the original trial was MAKE30, a composite of death from any cause, receipt of RRT, or persistent renal dysfunction within 30 days. No ethical approval was required for this secondary analysis; however, the SODa-BIC trial had ethical approval (HREC/89263/Alfred-2023) and was registered with ClinicalTrials.gov (NCT05697770). For the primary win ratio analysis, patients were included if data were available for all outcomes, consistent with previous trials.^(10,11)^

### Outcome definitions

For the win ratio analysis, outcomes were prioritized according to clinical importance using the following hierarchy: in-hospital death by Day 30; receipt of RRT within 30 days; persistent renal dysfunction within 30 days; and hospital length of stay (rounded to the nearest day). Persistent renal dysfunction was defined according to the original trial definition as a final inpatient creatinine value closest to Day 30 that was at least 200% of baseline. The hierarchy was determined by consensus among the study investigators, preserving the clinically accepted ordering of the MAKE30 components, with consideration of the clinical severity and patient relevance of each outcome, and with hospital length of stay included as the final tie-breaking outcome (Table 1S - Supplementary Material).

For the DOOR analysis, participants were assigned to one of five mutually exclusive categories ranked from least to most desirable outcome: in-hospital death by Day 30; in-hospital survival at Day 30 with both RRT and persistent renal dysfunction; in-hospital survival at Day 30 with RRT but without persistent renal dysfunction; in-hospital survival at Day 30 with persistent renal dysfunction but without RRT; and in-hospital survival at Day 30 without RRT or persistent renal dysfunction. Hospital length of stay was used as a tiebreaker for participants in the same DOOR category.

### Statistical analysis

Win ratio analyses were performed according to the method described by Pocock et al.^(7)^ Each participant in the Sodium Bicarbonate Group was compared with every participant in the Placebo Group, generating all possible treatment-control pairs. Within each pair, outcomes were assessed sequentially according to the predefined hierarchy until a winner was identified. If no difference was observed after evaluation of all hierarchical levels, the comparison was classified as a tie. This approach followed the conventional win ratio framework, maximizing the information available from each pair, and is consistent with previous applications of the win ratio. Given the potential issues surrounding comparisons after death, we also prespecified a sensitivity analysis in which pairs in which both patients died were considered tied and were not evaluated further.

The win ratio was calculated as the ratio of total wins to total losses. Values greater than one favored sodium bicarbonate, whereas values less than one favored placebo. Net treatment benefit was calculated as the difference between wins and losses divided by the total number of pairwise comparisons. Bootstrap resampling with 2,000 iterations was used to estimate 95% confidence intervals (95%CIs).^(9,11–13)^

The DOOR analyses were conducted according to established methodology.^(14,15)^ The Better DOOR Probability was calculated as the probability that a randomly selected participant assigned to sodium bicarbonate would have a more desirable outcome than a randomly selected participant assigned to placebo, with ties contributing half weight. A Better DOOR Probability of 0.50 indicates no treatment effect. Comparisons were made using the Mann-Whitney U test. Within comparisons, if a patient had a higher DOOR, then they received a higher rank.

Predefined subgroup analyses examined treatment effects according to baseline pH (< 7.25 *versus* ≥ 7.25), presence of stage 2 or 3 acute kidney injury (AKI), presence of septic shock, and type of metabolic acidosis (high anion gap versus non-anion gap). Interaction testing for the Win Ratio analyses was performed by comparing subgroup-specific log Win Ratios using a z-test.^(12)^

Sensitivity analyses included: exclusion of early ties occurring after death comparisons (if both patients in the pair died, they were excluded from the analyses at this point, and no further comparisons were made at lower levels of the hierarchy); application of a minimum three-day difference in hospital length of stay to define a win (if a pair was being compared at the length of stay level, then to record a win, the length of stay had to be at least three days shorter than the comparison);^(10)^ reversal of the hierarchy of RRT and persistent renal dysfunction given the potential long sequalae of AKI;^(16)^ and stratified win ratio analyses according to baseline sepsis status using Mantel-Haenszel-type weighting (this analysis was undertaken as a sensitivity analysis using a clinically important prognostic variable rather than to account for the randomisation strata of the parent trial).^(17)^ All analyses were performed using R version 4.3.3 (R Foundation for Statistical Computing, Vienna, Austria).

## RESULTS

Between April 2023 and December 2025, 500 patients were enrolled from 55 ICUs in seven countries. Following the withdrawal of consent by two patients, 498 patients were left in the modified intention-to-treat population. Baseline characteristics were similar between the two groups (Table 1). The study drug was administered in 99.4% of patients, and the median duration between randomization and commencement of study drug was 0.4 (interquartile range [IQR] 0.3 - 0.6) hour. At Day 30, 40.2% of the patients in the Sodium Bicarbonate Group and 39.4% in the Placebo Group had developed MAKE30 (Tables 2S and 3S - Supplementary Material).

**Table 1:** Baseline characteristics and outcomes of the included patients

		Sodium Bicarbonate (n = 244)	Placebo (n = 254)
Median age (years)		65.0 (51.8 - 74.0)	68.0 (55.2 - 76.0)
Female sex		111 (45.5)	104 (40.9)
Median minutes between eligibility and randomization		39.9 (15.0 - 73.3)	37.8 (12.0 - 70.6)
Type of admission			
	Medical	119 (48.8)	123 (48.4)
	Emergency surgery	64 (26.2)	66 (26.0)
	Elective surgery	61 (25.0)	65 (25.6)
Median APACHE II score on ICU admission*		21 (1626)	22 (17 - 27)
Median SOFA closest to randomization†		7 (6 - 9)	7 (6 - 9)
Cause of metabolic acidosis‡			
	Sepsis and septic shock	122 (50.0)	119 (46.9)
	Cardiogenic shock	40 (16.4)	56 (22.0)
	Hypovolemia/Dehydration	32 (13.1)	20 (7.9)
	Cardiac arrest	19 (7.8)	28 (11.0)
	Postoperative	9 (3.7)	15 (5.9)
	Vasoplegia	6 (2.5)	5 (2.0)
	Acute kidney injury§	3 (1.2)	5 (2.0)
	Hemorrhage	3 (1.2)	3 (1.2)
	Anaphylaxis and anaphylactic shock	1 (0.4)	1 (0.4)
	Other	9 (3.7)	2 (0.8)
Blood gas at eligibility			
	Median pH	7.26 (7.22 - 7.28)	7.26 (7.23 - 7.28)
	pH < 7.25	93 (38.1)	99 (39.0)
	Median base excess (mmol/L)	-9.2 (-11.5 - -7.0)	-8.8 (-11.0 - -7.0)
	Median PaCO_2_ (mmHg)	41 (35 - 44)	41 (36 - 44)
	Median bicarbonate (mmol/L)	17.0 (15.0 - 19.2)	18.0 (15.9 - 19.1)
Premorbid creatinine (mg/dL)¶		1.05 (0.80 - 1.40)	1.04 (0.81 - 1.41)
Pre-randomization			
	Median creatinine (mg/dL)	1.46 (1.07 - 2.18)	1.42 (1.05 - 2.14)
	Median lactate (mmol/L)	2.5 (1.4 - 4.5)	2.5 (1.5 - 4.5)
	Median sodium (mEq/L)	137 (133 - 140)	137 (134 - 139)
	Received sodium bicarbonate	35 (14.3)	23 (9.1)
	Acute kidney injury||	113/243 (46.5)	115/251 (45.8)
	Mechanical ventilation	191 (78.3)	207 (81.5)
	Sepsis#	163 (66.8)	174 (68.5)
	Septic shock#	95 (38.9)	104 (40.9)
	Median noradrenaline dose (μg/kg/min)	0.14 (0.07 - 0.23)	0.15 (0.07 - 0.23)

APACHE - Acute Physiology and Chronic Health Evaluation; ICU - intensive care unit; SOFA - Sequential Organ Failure Assessment; PaCO_2_: partial pressure of carbon dioxide in arterial blood.

* The Acute Physiologic Assessment and Chronic Health Evaluation II is a scoring system used in critically ill patients. Scores were calculated on the day of intensive care unit admission. Scores range from 0 to 71, with higher scores indicating a higher severity of illness and a greater likelihood of death in the hospital;

† the Sequential Organ Failure Assessment score is a scoring system used in critically ill patients. Scores range from 0 to 24, with higher scores indicating a higher severity of illness. This excludes the neurological component of Sequential Organ Failure Assessment;

‡ the presumed primary cause of metabolic acidosis was recorded by the bedside clinical team based on overall clinical assessment at the time of enrolment;

§ includes acute on chronic kidney disease;

¶ premorbid creatinine is the most recent serum creatinine measured within 3 months and more than 7 days before the intensive care unit admission. When not available, premorbid creatinine was imputed using the following formula: creatinine = 0.74 - 0.2 [if female] + 0.08 [if African American] + 0.003 × age [in years];

|| based on the creatinine criteria from KDIGO definition;

# sepsis defined as infection and total Sequential Organ Failure Assessment or modified Sequential Organ Failure Assessment ≥ 2. Septic shock defined as sepsis, use of vasopressor and lactate > 2 mmol/L. Data are median (interquartile range), n (%) or n/total (%).

### Win ratio

Data were missing for ten patients, being four in the Bicarbonate Group and six in the Placebo Group. Missingness related to persistent renal dysfunction occurred in four patients and hospital length of stay in six patients. Consequently, 488 patients (240 patients in the Bicarbonate Group and 248 in the Placebo Group) were included in the primary win ratio analysis, generating 59,520 pairwise comparisons.

The overall win ratio was 0.97 (95%CI 0.79 - 1.19), indicating no evidence that sodium bicarbonate improved the hierarchical composite outcome compared with placebo. Most pairwise comparisons were resolved at the mortality and hospital length of stay levels of the hierarchy, whereas relatively few were determined by persistent renal dysfunction. The distribution of wins, losses, and ties across hierarchical levels is shown in figure 1. The corresponding net treatment benefit was -1.7% (95%CI -11.7% - 8.7%). Results were consistent across all sensitivity analyses, including exclusion of early ties, application of a three-day threshold for hospital length of stay, reversal of the kidney-related components within the hierarchy, and stratification according to baseline sepsis status (Figure 2 and Figures 3S - 5S [Supplementary Material]).

**Figure 1:**
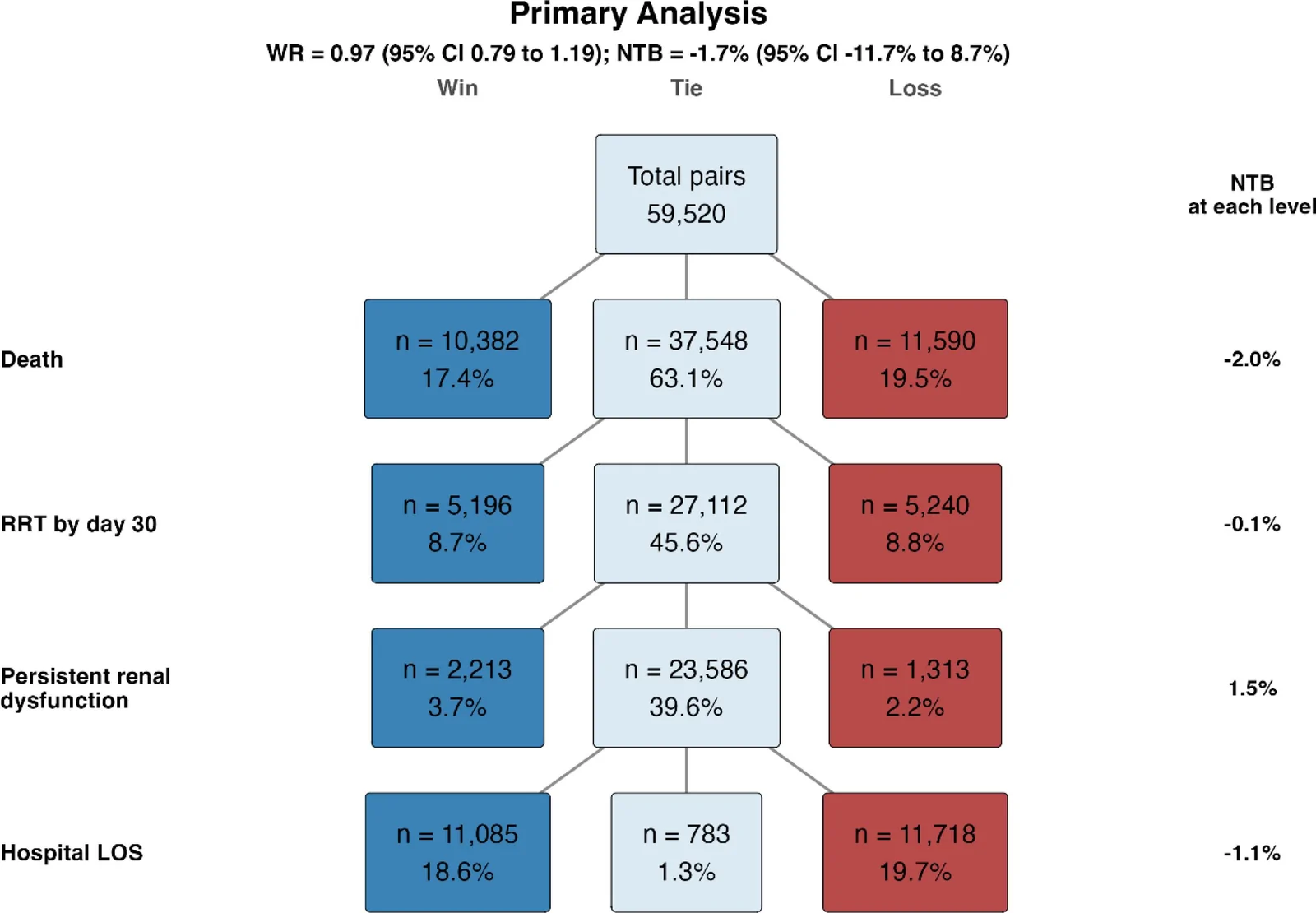
Win ratio hierarchy analysis of the primary outcome.

WR- win ratio; NTB - net treatment benefit, calculated as (wins - losses)/total number of pairwise comparisons; RRT - renal replacement therapy; LOS - length of stay.

Distribution of wins, losses, and ties across the hierarchical components of the primary win ratio analysis. For each patient with complete outcome data, those assigned to sodium bicarbonate were compared with every patient assigned to placebo, generating 59,520 pairwise comparisons. Outcomes were evaluated sequentially according to the predefined hierarchy of death by Day 30, receipt of renal replacement therapy by Day 30, persistent renal dysfunction by Day 30, and hospital length of stay. Comparisons not resolved at a higher level progressed to the next level of the hierarchy. The overall win ratio was 0.97 (95%CI 0.79 - 1.19), corresponding to a net treatment benefit of -1.7% (95%CI -11.7% - 8.7%). Percentages represent the proportion of all pairwise comparisons resolved at each level of the hierarchy.

**Figure 2:**
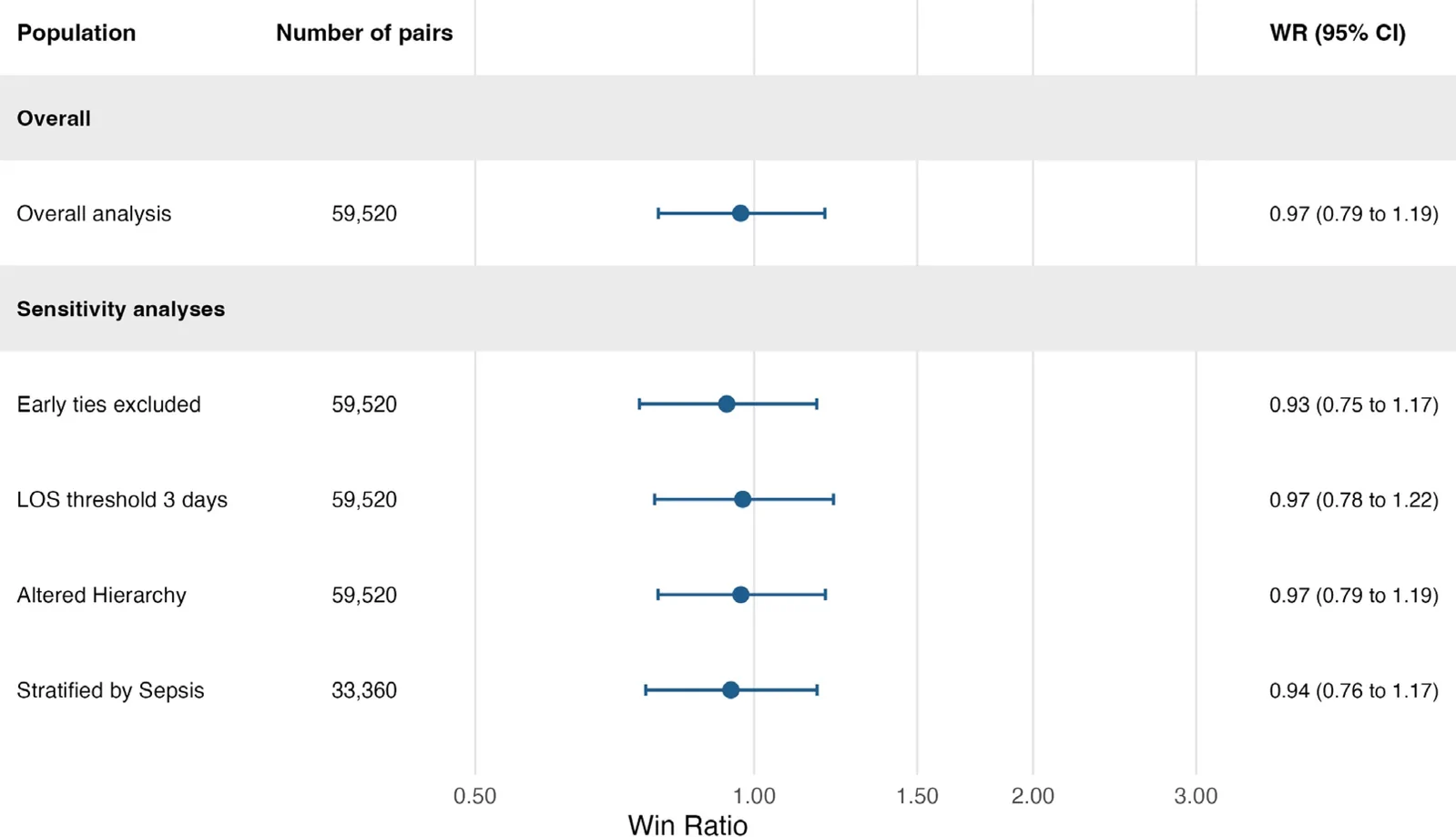
Sensitivity analyses of the win ratio.

WR - win ratio; LOS - length of stay.

Sensitivity analyses of the hierarchical composite outcome using the win ratio methodology. Analyses included exclusion of early ties, differing methods to assess missing data, application of a three-day threshold for hospital length of stay, altered hierarchies with use of renal replacement therapy duration as opposed to renal replacement therapy need (whereby patients who did not receive renal replacement therapy received a duration of 0 days) and reversal of persistent renal dysfunction and renal replacement therapy need in the hierarchy order, stratification according to baseline sepsis status. Points represent win ratio estimates and horizontal lines represent 95% confidence intervals. A win ratio greater than 1 favors sodium bicarbonate, whereas a win ratio less than 1 favors placebo. The vertical reference line at 1.0 represents no treatment effect.

### Subgroup analysis

Evidence of treatment-effect heterogeneity was observed according to baseline AKI status (*p* interaction = 0.010) and severity of acidemia (*p* interaction = 0.048) (Figure 3 and Figures 6S - 7S [Supplementary Material]). Patients with stage 2 or 3 AKI demonstrated a higher probability of favorable outcomes with sodium bicarbonate, whereas no such signal was observed among patients without AKI. In addition, the net treatment benefit at the mortality level was 21.0%; however, in the non-AKI subgroup, this was reversed to a level of -7.9%. No significant interactions were observed according to the presence of septic shock or the type of metabolic acidosis.

**Figure 3:**
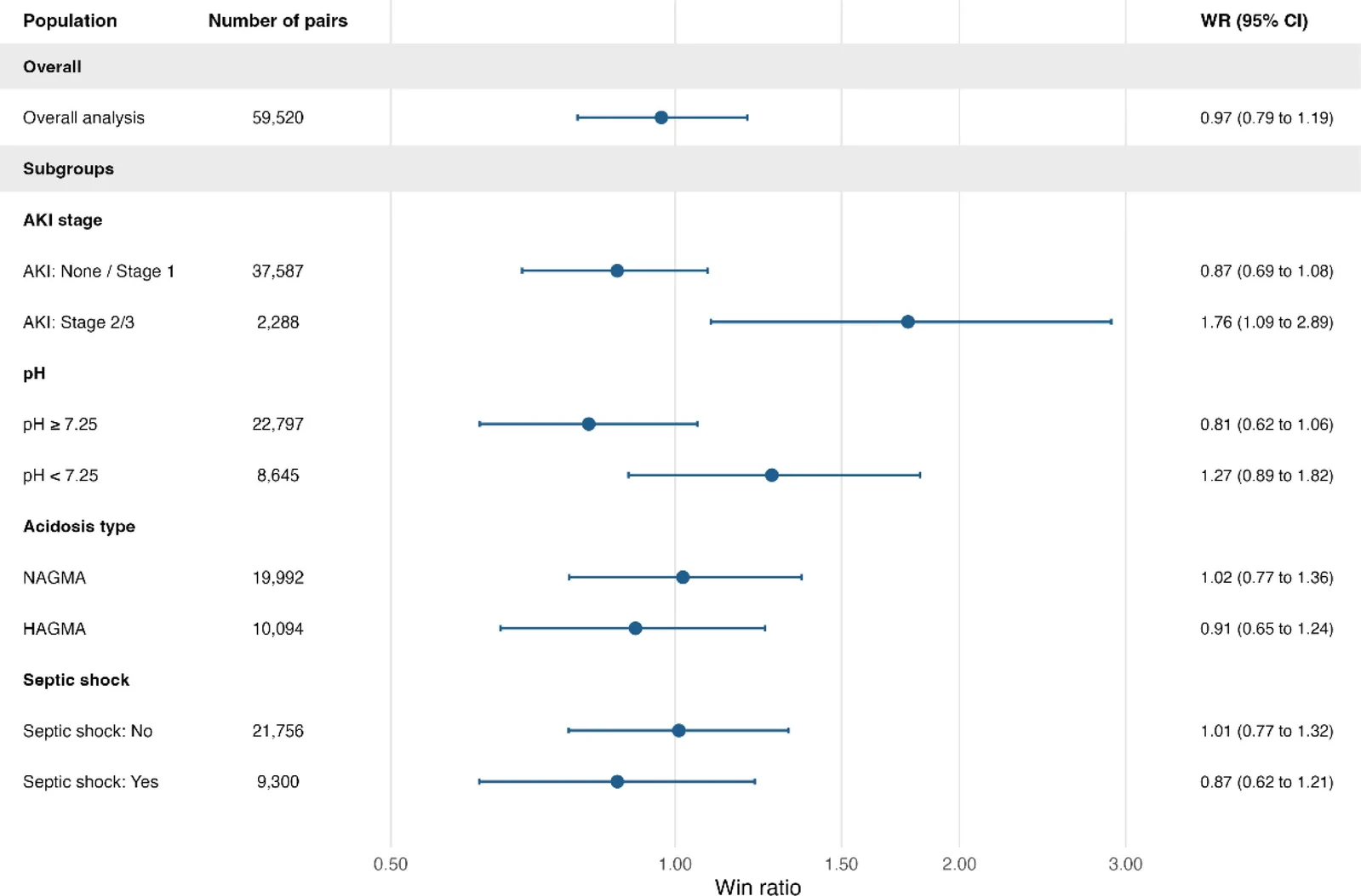
Subgroup analyses of the win ratio.

WR - win ratio; AKI - acute kidney injury; NAGMA - non-anion gap metabolic acidosis; HAGMA - high-anion gap metabolic acidosis.

Prespecified subgroup analyses of the hierarchical composite outcome using the win ratio methodology according to baseline acute kidney injury status, severity of acidemia, type of metabolic acidosis, and presence of septic shock. Points represent subgroup-specific win ratio estimates, and horizontal lines represent 95% confidence intervals. A win ratio greater than 1 favors sodium bicarbonate, whereas a win ratio less than 1 favors placebo. Interaction testing demonstrated evidence of treatment-effect heterogeneity according to acute kidney injury status (p for interaction = 0.01) and baseline pH category (p for interaction = 0.048), but not according to acidosis type (p for interaction = 0.597) or septic shock status (p for interaction = 0.494).

Acute kidney injury based on the creatinine criteria from Kidney Disease: Improving Global Outcomes (KDIGO) definition. Type of acidosis defined as high anion gap [> 12mEq/L] *versus* non-anion gap [≤ 12mEq/L]. Septic shock was defined as sepsis requiring vasopressor therapy and lactate > 2mmol/L.

## DOOR

The Better DOOR Probability was 0.49 (95%CI 0.45 - 0.54), indicating no overall difference in the probability of achieving a more desirable outcome with sodium bicarbonate compared with placebo (Figure 4). Similar results were observed when hospital length of stay was incorporated as a tiebreaker. Approximately 60% of participants in both groups were classified in the most favorable DOOR category, defined as survival without RRT or persistent renal dysfunction (Figure 8S - Supplementary Material). Among prespecified subgroups, only patients with stage 2 or 3 AKI demonstrated a signal favoring sodium bicarbonate, with a Better DOOR Probability of 0.62 (95%CI 0.51 - 0.73).

**Figure 4:**
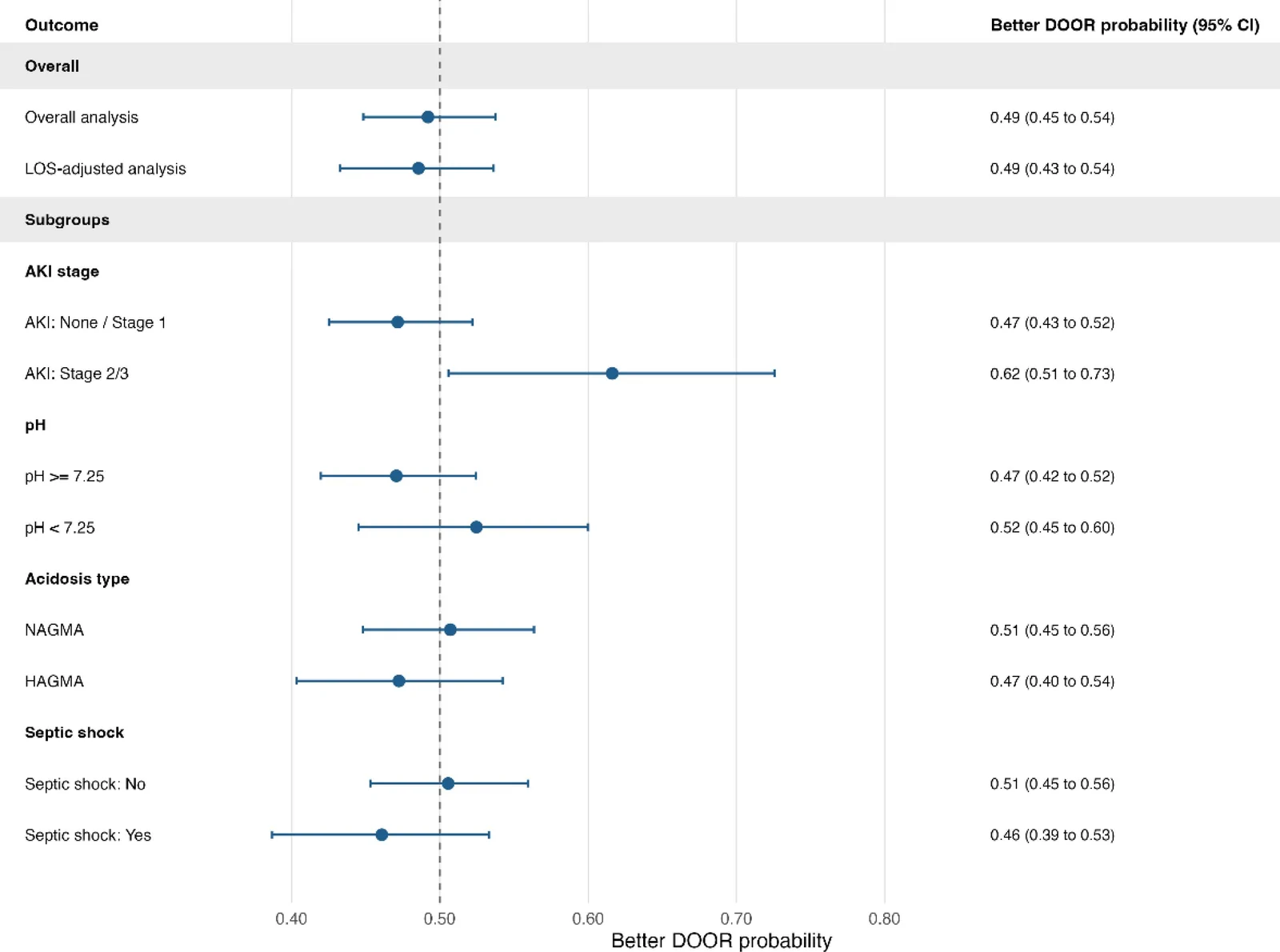
Desirability of Outcome Ranking (DOOR) analysis.

DOOR - Desirability of Outcome Ranking; LOS - length of stay; AKI - acute kidney injury; NAGMA - non-anion gap metabolic acidosis; HAGMA - high-anion gap metabolic acidosis.

Better DOOR probability estimates overall and within prespecified subgroups. The Better DOOR probability represents the probability that a randomly selected patient receiving sodium bicarbonate would have a more desirable overall outcome than a randomly selected patient receiving placebo. Points represent Better DOOR probability estimates, and horizontal lines represent 95% confidence intervals. A Better DOOR probability of 0.50 indicates no treatment effect. Values greater than 0.50 favor sodium bicarbonate, whereas values less than 0.50 favor placebo. The overall Better DOOR probability was 0.49 (95%CI 0.45 - 0.54). DOOR categories were defined according to day-30 survival status, receipt of renal replacement therapy, and persistent renal dysfunction.

Acute kidney injury based on the creatinine criteria from Kidney Disease: Improving Global Outcomes (KDIGO) definition. Type of acidosis defined as high anion gap [> 12mEq/L] vs. non-anion gap [≤ 12mEq/L]. Septic shock was defined as sepsis requiring vasopressor therapy and lactate > 2 mmol/L.

## DISCUSSION

In this post hoc analysis of the SODa-BIC trial, the use of two hierarchical composite outcome approaches, the win ratio and DOOR, did not alter the overall interpretation of the original trial. Using both analysis methods, sodium bicarbonate was not associated with an overall improvement in patient-centered outcomes. However, these analyses provided additional insight into the relative contribution of individual outcome components and identified signals of potential heterogeneity of treatment effect that were less apparent in the conventional MAKE30 analysis.

The primary outcome of MAKE30 is a composite outcome and, while such composite outcomes improve statistical efficiency, they assume that all components are of equal importance.^(18)^ In contrast, hierarchical approaches prioritize clinically important events, such as death, before evaluating less severe outcomes.^(9)^ Despite this difference in analytical framework, both the win ratio and DOOR analyses produced findings that were consistent with the original trial, supporting the robustness of the neutral primary result.

Although no overall treatment effect was observed, subgroup analyses suggested that sodium bicarbonate may benefit patients with stage 2 or 3 AKI and those with more severe acidemia. These findings are biologically plausible^(19,20)^ and consistent with previous studies in which sodium bicarbonate appeared to reduce kidney-related complications among patients with more severe metabolic derangements.^(2,3)^ While these observations should be interpreted cautiously given the post hoc nature of the analyses, they provide additional support for the hypothesis that treatment effects may vary according to baseline kidney injury and severity of acidosis.

An important observation was that relatively few pairwise comparisons were resolved at the level of persistent renal dysfunction; most were determined by mortality and hospital length of stay. This finding suggests that, within this population, persistent renal dysfunction contributed less to overall outcome discrimination than anticipated and highlights how hierarchical approaches can provide insight into the relative influence of individual components within a composite endpoint. However, the contribution of individual components to a hierarchical composite outcome reflects not only their clinical importance but also their frequency, distribution between treatment groups, and position within the prespecified hierarchy. Accordingly, the relatively small contribution of persistent renal dysfunction should not be interpreted as indicating that this outcome is of limited clinical importance. Such information is not readily available from conventional composite outcome analyses.^(21)^ In addition, it is plausible that kidney effects may in part be related to direct pH effects altering clinician behavior in relation to RRT initiation as opposed to an effect on the severity of kidney damage or recovery.

The ability of the win ratio to integrate continuous and binary outcomes, unlike a traditional composite, may enable the inclusion of outcomes that allow for better discrimination. For example, the change in glomerular filtration rate has been proposed in trials assessing chronic kidney disease,^(22)^ and these changes over an extended period have been associated with long-term mortality.^(23)^ Acute kidney injury has notable long-term sequelae,^(16,24)^ and as such, the use of a win ratio may allow for a more nuanced understanding of the trajectory of AKI and the effect of interventions as opposed to a discrete, binary outcome assessing persistent renal dysfunction at a fixed time-point.

The comparison of win ratio and DOOR methodologies also demonstrated substantial consistency. Although these approaches differ conceptually, both identified no overall treatment effect, and both suggested a potential signal of benefit among patients with stage 2 or 3 AKI. This concordance increases confidence that the observed findings are not specific to a particular analytical framework and supports the use of hierarchical approaches as complementary methods for analyzing complex outcomes in critical care trials. For instance, the win ratio can only consider one outcome at each level, whereas DOOR can integrate multiple events into each mutually exclusive hierarchical level.^(25)^ Despite the increasing use of the win ratio in critical care trials, those using a DOOR outcome have been largely limited to other specialties such as infectious diseases.^(25,26)^ Additionally, their use may reduce over-interpretation of secondary outcomes, which may be subject to issues of competing risk.

This study has several limitations. First, all analyses were conducted post hoc and should therefore be considered exploratory. Second, the trial was not powered for subgroup analyses, increasing the possibility that observed interactions occurred by chance. Third, the choice and ordering of hierarchical outcome components was inherently subjective and alternative hierarchies may have produced different estimates. Fourth, although continuing pairwise comparisons beyond death follows the conventional win ratio methodology, some may consider treating patients who both die as tied to be more clinically intuitive. Reassuringly, our findings were consistent using both approaches. Finally, although hierarchical approaches improve clinical interpretability, they do not overcome limitations related to sample size or residual uncertainty surrounding treatment effects.

## CONCLUSION

In this secondary analysis of the SODa-BIC trial, hierarchical composite outcome analyses using Win Ratio and DOOR methodologies were consistent with primary trial analyses and showed no evidence of an overall benefit of sodium bicarbonate. However, these approaches provided additional insight into the contribution of individual outcome components and suggested patients with more severe acute kidney injury and academia may benefit from sodium bicarbonate therapy. Hierarchical outcome methods may complement conventional composite outcome analyses in future critical care trials.

## Data Availability

The contents of this manuscript relate to a previously published trial, and access to this data. For access to the data, please refer to the data sharing statement published with the original SODa-BIC trial.
