# Supplementary material for: A secondary analysis of the SODa-BIC randomized clinical trial using multiple hierarchical composite outcomes
Source: Crit Care Sci. 2026 Sep 17;38:e20260279. doi: 10.62675/2965-2774.20260279 (PMC13690031; doi:10.62675/2965-2774.20260279)
Supplement: Supplementary file 1 [file 2965-2774-ccsci-38-e20260279-Suppl01.pdf]

Critical Care Science
https://doi.org/10.62675/2965-2774.20260279

SUPPLEMENTARY MATERIAL

A secondary analysis of the SODa-BIC randomized clinical trial using multiple hierarchical composite outcomes

Humphrey G. M. Walker[1,2,3], Alastair Brown[1,3,4], Kyle C. White[5,6], Mairead McNamara[4], D. Jamie Cooper[4,7], Tomoko Fujii[4,8], Alisa M. Higgins[4], Carol L. Hodgson[3,4,7], Leanlove Navarra[9,10], Alistair Nichol[4,7,11], Sandra Peake[4,12,13], Alvaro Rea-Neto[14], Paul Secombe[4,15,16], Fiona Osborne[4], Hugh Burrill[4], Emily See[3,17], Meredith Young[7], Fernando Godinho Zampieri[18], Mark Plummer[3,19,20], Connor Christie[20], Leo Nunnink[6,21], Josephine Mackay[6], Shay McGuinness[22], Joradee Marmol[9], Ravindranath Tiruvoipati[23,24,25], Michael Bailey[4,16], Paul J. Young[3,4,9,10], Andrew Udy[4,7], Ary Serpa Neto[3,4,26,27]

THE SODA-BIC INVESTIGATORS

Australia

Austin Hospital: Ary Serpa Neto, Rinaldo Bellomo, Glenn Eastwood, Leah Peck, Helen Young, Alessandro Caroli, Jonathan Nübel, Nuanprae Kitisin, Nuttapol Pattamin, Yukiko Hikasa, Akinori Maeda, Sofia Spano, Anis Chaba
Bendigo Health: Cameron Knott, Angus Carter, Vijay Palaniswamy, Julie Smith
Bunbury Regional Hospital: Renesh Nair, Sharon Waterson
Cairns Hospital: Rajendra Goud, Sarah Furness, Rahul Tambade, Craig McDonald, Richard Mooney, Seb Blank, Alison Main, Mitch Cameron, Pieter Weemaes
Calvary Mater Newcastle: Samara Pandhem, Laurence Emery
Canberra Hospital: Ramprasad Vegunta, Kiran Gudivada, Sumeet Rai, Susan Donnelly, Tash Edmunds, Mary Nourse, Grainne Hughes, Lidia Zec
Casey Hospital: Umesh Kadam, Sandra Cork, Seema Radhakrishnan
Dandenong Hospital: Umesh Kadam, Ashwin Subramaniam, Daniel Hwang
Epworth: Kyle Brooks, Gabrielle Hanlon, Laurie Saffer, Judith Watson
Flinders Medical Centre: Shailesh Bihari, Alpesh Patel, JoAnne McIntyre
Footscray Hospital: Craig French, Yang Yang, James Douglas, Forbes McGain, Samantha Bates, Miriam Towns, Haindavi Muppa, Stephanie Menadue, Stephanie Schembri, Anna Truong, Vivian Nguyen, Susie Peace, Abi Osman
Frankston Hospital: Sachin Gupta, Ravindranath Tiruvoipati, David Zhang
Gold Coast University Hospital: James McCullough, Maree Houbert, Julie Pitman, Mandy Tallott, Jennie Walker
Gosford Hospital: Atul Gaur, Shelley Donovan, Eloise Hair, Elisha Turner, Christine Bailey, Saara Mohamed, Niyaz Ashraf, Cyril Tang, Max McGill, Wendy Elliss, Pavani Wettasinghe, Avadhut Zagade, Lauren Franks, David Wallace, Taylor Beatty, Charles Wilmot, Phillip St Flour, Patrick O'Connell
Grampians Health: Khaled El-Khawas, Mark Kubicki, Dianne Brown, Andrea Kramer
Ipswich Hospital: Neeraj Bhadange, Vijo Kuruvilla, Hannah McCabe, Wayne Stevens
Lyell McEwin Hospital: Vishwanath Biradar, Natalie Soar, Natalie Bennett, Katherine Ye, Ryan Sachs, Bec Martin, Laura Hall
Mater Health: Ravi Chockalingam Pillai, Isabel Anne Leditschke, Loki Johnk, Alexander Tait, Katherine Jongebloed-Sadam, Gemma Hammond, Amar Pandey, Fraser Morris, Vishal Narwade
Monash Medical Centre: Yahya Shehabi, Neil Glassford, Alice Li**,** Siew Wai Wong
Nepean Hospital: Ian Seppelt, Idunn Morris, Rebecca Gresham, Sheeja Joy, Julie Lowrey, Kristy Masters, Narges Niya
Orange Health Service: James Basha, Alic Henschke, Renae Bryant, Merryn Jones

This is an open access article under the CC BY license (https://creativecommons.org/licenses/by/4.0/).

Peninsula Private Hospital: Ravindranath Tiruvoipati, Hannah Rule, David McArdle, Fatima Husaini, Emma Toulmin, Monika Shrestha
Princess Alexandra Hospital: Kyle White, Leo Nunnink, Megan Davis, Meg Harward, Kylie O'Neill, Josephine Mackay, Jason Meyer
Queen Elizabeth II Jubilee Hospital: Ahmad Nasser, David Stewart, Kunjal Patel
Royal Adelaide Hospital: Mark Plummer, Paola Acre-Arango, Nerissa Brown, Connor Christie, Sarah Doherty, Kathleen Glasby, Justine Rivett, Imogen Asser, Mahmoud Idris
Royal Brisbane and Women's Hospital: Elissa Milford, Cheryl Fourie, Marianne Kirrane, Melissa Lassig-Smith, Amelia Livermore, Janine Stuart
Royal Darwin Hospital: Lewis Campbell, Kate Babbage, Mary Ann Tiras
Royal North Shore Hospital: Chris Andersen, Anthony Delaney, Frances Bass, Rosalind Elliott, Emily Fitzgerald, Naomi Hammond, Tessa Garside, Lachlan Donaldson, Anne O'Connor, Elizabeth Yarad
Royal Perth Hospital: Yves Ineza, Trisha Hardman, Sharon Waterson
Royal Prince Alfred Hospital: Claire Seiffert, David Gattas, Elliott Worku, Mathew Phillips, Heidi Buhr, Bronwyn Kelly, Cindy Liang
St Vincent's Hospital Melbourne: Alastair Brown, Humphrey Walker, Tim Haydon, Jennifer Holmes, Melissa King, Viean Luk, Adelle Ordering, Espedito Faraone
St Vincent's Hospital Sydney: Priya Nair, Amelia Hall, Sarah Henstridge, Amelia Liu, Sally Newman, Alexandra Campion
Sunshine Coast University Hospital: Victoria Campbell, Lauren Murray, Peter Garrett, Jane Brailsford, Moreblessing Kajevu
Sunshine Hospital: Craig French, Yang Yang, James Douglas, Forbes McGain, Samantha Bates, Miriam Towns, Haindavi Muppa, Stephanie Menadue, Stephanie Schembri, Anna Truong, Vivian Nguyen, Susie Peace, Abi Osman
Sydney Adventist Hospital: Tessa Garside, Celia Bradford, Alison Scott, Linley Shields, Susan Haworth, Emma Smith, Stephanie Kirwan, Kim Hubbard, Minhee Chan, Georgia Hammett
The Alfred Hospital: Andrew Udy, Meredith Young, Jasmin Board, Emma-Leah Martin, Laura Adams
The Queen Elizabeth Hospital: Sandra Peake, Nikki Yeo, Patricia Williams, Catherine Kurenda, Srilatha Vemparala, Danila Bigiolli
The Sutherland Hospital: Anas Naeem, Kesley Matthews, Elizabeth Turner
Townsville University Hospital: Sananta Dash, Karen Carson, Monica Watson
University Hospital Geelong: Erfana Thashneem, Neil Orford, Simone Fitzgerald, Stacey Hawker, Jemma Trickey
Victorian Heart Hospital: Wisam Al-Bassam, Roger Smith

New Zealand

Auckland CVICU: Shay McGuiness, Magdalena Butler, Keri-Anne Cowdrey, Alix Gray, Rachael Parke, Melissa Robertson, Laura Weiss, Brittany Mason
Auckland DCCM: Chris Hands, Caroline O'Connor, Hiromi Nakamuro, Archie Maclang, Lizzie Briggs, Kristian Misa, Michael Lozano
Christchurch Hospital: Toby Betteridge, Jan Mehrtens, Anna Morris
Dunedin Hospital: Pawel Twardowski, Katherine Perry, Martin Jones, Hans Waibel, Markus Renner, Martin Dvoracek, Galel Yakobi, Kate Stephens, David Silverman, Myles Smith, Bobbi Butler, Alisha Da Silva, Christina Kumate, Jordan McGregor, Sarah Taylor
Middlemore Hospital: Tony Williams, Michael Borrie, Tina Mwembani, Rima Song, Vivian Lai, Katrina Medina
Rotorua Hospital: Arihia Waaka, Gareth Fowler, Cornelius Van Wyk, Luke Ferrari, Siew-Bek Goh-Robinson
Waikato Hospital: Geoff McCracken, Fiona Gray, Jenny Karp, Mark Tan
Wellington Regional Hospital: Paul Young, Odessa David, Kirsha Delaney, Macaulay Diment, Reece Latonio, Joradee Marmol, Leanlove Navarra, Keh Ong, Nina Roberts, Shan Qiu

Japan

Jikei University Hospital: Tomoko Fujii, Shoki Notsu, Kosuke Yagi, Yoko Yamaguchi, Akira Kageyama, Kayo Okawa, Kanako Watanabe, Minako Adachi, Aya Watanabe, Kazuhiro Okada, Dai Harada, Shunsuke Hitosugi, Naomi Ando, Yumi Nakamura

India

Jawaharlal Institute of Postgraduate Medical Education & Research: Anusha Cherian
Deep Hospital: Kanwalpreet Sodhi, Rahul Kohli, Davinder Singh, Manender Kumar, Jinderjot Singh

Oman

Sultan Qaboos Comprehensive Cancer Care and Research Centre: Sulaiman Al Maqbali, Airton Leonardo De Oliveira Manoel, Marwan Al Abidi, Aida Malik AlKindy, Fatma Al Farsi, Najla AlHashmi

Saudi Arabia

King Abdullah International Medical Research Center: Yaseen Arabi, Fahad Al Rubayan, Laila Adawi, Asya Hawsa, Badriah Alanazi, Norah Alageel, Ahmad Alasmari, Abdullah Altamimi, Deema Algoblan, Abdulmajeed Alshammari, Musharaf Sadat, Asma Alanazi, Felwa Bin Humaid, Yasir Alzoubi, Haifa Alhumedi, Maram Aldosari, Asma Alzahrani, Nour Elsokby, Jawaher Alanazi, Ali Alshahrani, Sahar Alharbi, Amira Nasser Alsaleh, Haneen Sindi, Hadeel Alharbi, Raed Almalki, Reem Alsaif, Fay Almadhi, Rawan Osama Alkotop, Bader Alrufaiq

Brazil

Centro de Estudos e de Pesquisas em Terapia Intensiva: Álvaro Réa-Neto, Verônica Barros, Amanda Kozesinski, Jeane Vieira

TRIAL SPONSOR, COMMITTEES, FUNDING BODY AND ENDORSEMENT

Sponsor

Australian and New Zealand Intensive Care Research Centre (ANZIC-RC), Australia

Writing Committee

Ary Serpa Neto (Chair), Mairead McNamara, Kyle White, D. Jamie Cooper, Tomoko Fujii, Alisa M. Higgins, Carol L. Hodgson, Leanlove Navarra, Alistair Nichol, Sandra Peake, Alvaro Rea-Neto, Paul Secombe, Fiona Osborne, Hugh Burrill, Emily See, Meredith Young, Fernando Godinho Zampieri, Mark Plummer, Connor Christie, Leo Nunnink, Josephine Mackay, Shay McGuinness, Joradee Marmol, Ravindranath Tiruvoipati, Michael Bailey, Paul J. Young, Rinaldo Bellomo, Andrew Udy

Management Committee

Ary Serpa Neto (Chair), Mairead McNamara, Kyle White, D. Jamie Cooper, Tomoko Fujii, Alisa M. Higgins, Carol L. Hodgson, Leanlove Navarra, Alistair Nichol, Sandra Peake, Alvaro Rea-Neto, Paul Secombe, Emily See, Pam Taylor, Meredith Young, Fernando G Zampieri, Paul J. Young, Rinaldo Bellomo, Andrew Udy

Independent Data Safety Monitoring Committee

John Kellum – Pittsburgh, USA (Chair); Lakhmir Chawla – San Diego, USA; Anais Charles-Nelson – Melbourne, Australia

Funding bodies

The study was funded by a project grant from the National Health and Medical Research Council of Australia (Project Grant ID 2014960).
Jamie Cooper, Carol Hodgson and Rinaldo Bellomo are supported by Investigator Leadership Grants from the National Health and Medical Research Council of Australia (NHMRC), and Emily See and Alissa Higgins are supported by an NHMRC Emerging Leader Investigator Grants.
Paul Young was supported by a Health Research Council of New Zealand Clinical Practitioner Research Fellowship.

Endorsement

The study was endorsed by the Australian and New Zealand Intensive Care Society Clinical Trials Group.

ACKNOWLEDGEMENTS

We sincerely thank the patients and their families for their trust and participation. We are deeply grateful to the doctors, nurses, allied health professionals, and research coordinators whose dedication and collaboration made this trial possible. The authors and the ANZICS CORE management committee would like to thank clinicians, data collectors and researchers at the following contributing sites in Australia and New Zealand: Albury Base Hospital ICU, Alfred Hospital ICU, Alice Springs Hospital ICU, Angliss Hospital ICU, Armadale Health Service ICU, Ashford Community Hospital ICU, Auckland City Hospital CV ICU, Auckland City Hospital DCCM, Austin Hospital ICU, Ballarat Health Services ICU, Bankstown-Lidcombe Hospital ICU, Bathurst Base Hospital ICU, Bendigo Health Care Group ICU, Blacktown Hospital ICU, Bowral Hospital HDU, Box Hill Hospital ICU, Brisbane Private Hospital ICU, Broken Hill Base Hospital; Health Services ICU, Buderim Private Hospital ICU, Bunbury Regional Hospital ICU, Bundaberg Base Hospital ICU, Caboolture Hospital ICU, Cabrini Hospital ICU, Cairns Hospital ICU, Calvary Bruce Private Hospital HDU, Calvary Hospital (Canberra) ICU, Calvary Hospital (Lenah Valley) ICU, Calvary John James Hospital ICU, Calvary Mater Newcastle ICU, Calvary North Adelaide Hospital ICU, Calvary Wakefield Hospital (Adelaide) ICU, Campbelltown Hospital ICU, Canberra Hospital ICU, Casey Hospital ICU, Central Gippsland Health Service ICU, Christchurch Hospital ICU, Coffs Harbour Health Campus ICU, Concord Hospital (Sydney) ICU, Dandenong Hospital ICU, Dubbo Base Hospital ICU, Dunedin Hospital ICU, Echuca Regional Hospital HDU, Epworth Eastern Private Hospital ICU, Epworth Freemasons Hospital ICU, Epworth Geelong ICU, Epworth Hospital (Richmond) ICU, Fairfield Hospital ICU, Fiona Stanley Hospital ICU, Flinders Medical Centre ICU, Flinders Private Hospital ICU, Footscray Hospital ICU, Frankston Hospital ICU, Gold Coast Private Hospital ICU, Gold Coast University Hospital ICU, Gosford Hospital ICU, Gosford Private Hospital ICU, Goulburn Base Hospital ICU, Goulburn Valley Health ICU, Grafton Base Hospital ICU, Greenslopes Private Hospital ICU, Griffith Base Hospital ICU, Hawkes Bay Hospital ICU, Hervey Bay Hospital ICU, Hollywood Private Hospital ICU, Holmesglen Private Hospital ICU, Holy Spirit Northside Hospital ICU, Hornsby Ku-ring-gai Hospital ICU, Hurstville Private Hospital ICU, Hutt Hospital ICU, Ipswich Hospital ICU, John Fawkner Hospital ICU, John Flynn Private Hospital ICU, John Hunter Hospital ICU, Joondalup Health Campus ICU, Kareena Private Hospital ICU, Knox Private Hospital ICU, Latrobe Regional Hospital ICU, Launceston General Hospital ICU, Lingard Private Hospital ICU, Lismore Base Hospital ICU, Liverpool Hospital ICU, Logan Hospital ICU, Lyell McEwin Hospital ICU, Mackay Base Hospital ICU, Macquarie University Private Hospital ICU, Maitland Hospital HDU/CCU, Maitland Private Hospital, Manly Hospital & Community Health ICU, Manning Rural Referral Hospital ICU, Maroondah Hospital ICU, Mater Adults Hospital (Brisbane) ICU, Mater Health Services North Queensland ICU, Mater Private Hospital (Brisbane) ICU, Mater Private Hospital (Sydney) ICU, Melbourne Private Hospital ICU, Middlemore Hospital ICU, Mildura Base Hospital ICU, Monash Medical Centre-Clayton Campus ICU, Mount Hospital ICU, Mount Isa Hospital ICU, National Capital Private Hospital ICU, Nelson Hospital ICU, Nepean Hospital ICU, Nepean Private Hospital ICU, Newcastle Private Hospital ICU, Noosa Hospital ICU, North Shore Hospital ICU, North Shore Private Hospital ICU, North West Private Hospital ICU, North West Regional Hospital (Burnie) ICU, Northeast Health Wangaratta ICU, Northern Beaches Hospital, Norwest Private Hospital ICU, Orange Base Hospital ICU, Peninsula Private Hospital ICU, Pindara Private Hospital ICU, Port Macquarie Base Hospital ICU, Prince of Wales Hospital (Sydney) ICU, Prince of Wales Private Hospital (Sydney) ICU, Princess Alexandra Hospital ICU, Queen Elizabeth II Jubilee Hospital ICU, Redcliffe Hospital ICU, Robina Hospital ICU, Rockhampton Hospital ICU, Rockingham General Hospital ICU, Rotorua Hospital ICU, Royal Adelaide Hospital ICU, Royal Brisbane and Women's Hospital ICU, Royal Darwin Hospital ICU, Royal Hobart Hospital ICU, Royal] Melbourne Hospital ICU, Royal North Shore Hospital ICU, Royal Perth Hospital ICU, Royal Prince Alfred Hospital ICU, Ryde Hospital & Community Health Services ICU, Shoalhaven Hospital ICU, Sir Charles Gairdner Hospital ICU, South East Regional Hospital ICU, South West Healthcare (Warrnambool) ICU, Southern Cross Hospital (Wellington) ICU, Southland Hospital ICU, St Andrew's Hospital (Adelaide) ICU, St Andrew's Hospital Toowoomba ICU, St Andrew's Private Hospital (Ipswich) ICU, St Andrew's War Memorial Hospital ICU, St George Hospital (Sydney) ICU, St George Private Hospital (Sydney) ICU, St John of God (Berwick) ICU, St John Of God Health Care (Subiaco) ICU, St John Of God Hospital (Ballarat) ICU, St John of God Hospital (Bendigo) ICU, St John Of God Hospital (Geelong) ICU, St John Of God Hospital (Murdoch) ICU, St John of God Midland Public; Private ICU, St Vincent's Hospital (Melbourne) ICU, St Vincent's Hospital (Sydney) ICU, St Vincent's Hospital (Toowoomba)

ICU, St Vincent's Private Hospital (Sydney) ICU, St Vincent's Private Hospital Fitzroy ICU, Sunnybank Hospital ICU, Sunshine Coast University Hospital ICU, Sunshine Coast University Private Hospital ICU, Sunshine Hospital ICU, Sutherland Hospital; Community Health Services ICU, Sydney Adventist Hospital ICU, Sydney Southwest Private Hospital ICU, Tamworth Base Hospital ICU, Taranaki Health ICU, Tauranga Hospital ICU, The Bays Hospital ICU, The Chris O'Brien Lifehouse ICU, The Memorial Hospital (Adelaide) ICU, The Northern Hospital ICU, The Prince Charles Hospital ICU, The Queen Elizabeth (Adelaide) ICU, The Townsville Hospital ICU, The Valley Private Hospital ICU, The Wesley Hospital ICU, Timaru Hospital ICU, Toowoomba Hospital ICU, Tweed Heads District Hospital ICU, University Hospital Geelong ICU, Victorian Heart Hospital ICU, Wagga Wagga Base Hospital & District Health ICU, Waikato Hospital ICU, Wairau Hospital ICU, Warringal Private Hospital ICU, Wellington Hospital ICU, Werribee Mercy Hospital ICU, Western District Health Service (Hamilton) ICU, Western Hospital (SA) ICU, Western Private Hospital ICU, Westmead Hospital ICU, Westmead Private Hospital ICU, Whakatane Hospital ICU, Whangarei Area Hospital, Northland Health Ltd ICU, Wimmera Health Care Group (Horsham) ICU, Wollongong Hospital ICU, Wollongong Private Hospital ICU, Women's and Children's Hospital PICU, Wyong Hospital ICU.

ADMINISTRATIVE INFORMATION

Trial registration

The SODium BICarbonate for Metabolic Acidosis in the Intensive Care Unit (SODa-BIC) study was registered on clinicaltrials.gov before patient enrolment commenced (NCT05697770).

Statistical analysis plan

The SODa-BIC study was analyzed in accordance with a single prespecified statistical analysis plan published in Critical Care and Resuscitation before patient enrolment concluded (Protocol and Statistical Analysis Plan available at NEJM.org).[1]

Data and Safety Monitoring Committee

An independent Data and Safety Monitoring Committee (DSMC) conducted one formal interim analysis on 6 June 2025, when 125 patients (25%) had completed 90-day follow-up. The DSMC recommended the study continue with no changes to the protocol. The DSMC also conducted a prespecified blinded sample size re-estimation after enrolment of the first one hundred patients and recommended the study continue with the original planned sample size (the plan for the sample size re-estimation is described below).

Data sharing

The SODa-BIC study investigators will share the study data in a manner consistent with the Data Sharing Policy of Australian and New Zealand Intensive Care Research Centre (available at https://www.monash.edu/medicine/sphpm/anzicrc) which governs appropriate and responsible sharing of, and access to the Centre's data for scientific research.

Artificial Intelligence disclosure

Artificial Intelligence tools (ChatGPT) were used solely for language editing and improvement of readability during manuscript preparation. No statistical analyses, data interpretation, or scientific conclusions were generated using AI tools.

ADDITIONAL INFORMATION REGARDING THE PROTOCOL

Eligibility criteria

Inclusion criteria

1. Adult patients (≥ 18 years); and

2. Receiving a continuous infusion of a vasopressor (noradrenaline, adrenaline, vasopressin, phenylephrine, dopamine, angiotensin II, metaraminol and/or terlipressin) to maintain mean arterial pressure > 65mmHg (or as determined by the treating clinician); and

3. Dedicated line (central or peripheral) is available (or insertion is planned within the hour after randomization); and

4. Metabolic acidosis (within the last two hours before randomization):

pH < 7.30; and
Base excess ≤ -4mEq/L; and
Partial pressure of carbon dioxide in arterial blood ($PaCO_2$) ≤ 45mmHg (non-intubated) OR ≤ 50mmHg (intubated).
All the diagnostic criteria of metabolic acidosis had to be fulfilled within the two hours prior to randomization, and a vasopressor had to be continuously infused at the time of randomization.

Exclusion criteria

1. Fulfilled all eligibility criteria greater than 48 hours ago; OR

2. Suspected clinically significant digestive or urinary tract loss of sodium bicarbonate (e.g., diarrhea, ileostomy losses, renal tubular acidosis, or drainage of pancreatic or bile duct); OR

3. Diabetic ketoacidosis; OR

4. Estimated glomerular filtration rate < 30mL/min due to chronic kidney disease; OR

5. Currently receiving sodium bicarbonate at the moment of randomization (doses of sodium bicarbonate prior to randomization are allowed); OR

6. Currently receiving renal replacement therapy (acute or chronic) or planned to start renal replacement therapy in the next three hours (according to the treating clinical team); OR

7. Severe dysnatremia (serum Na ≥ 155mEq/L or < 120mEq/L); OR

8. Hypokalemia (serum K < 2.5mEq/L); OR

9. Pulmonary edema with PaO_2 / FiO_2 < 100; OR

10. Hypocalcemia (iCa < 0.8mmol/L); OR

11. Patients admitted to the ICU after a drug overdose or intoxication (including alcohol intoxication); OR

12. Pregnancy or breastfeeding; OR

13. Death is deemed to be inevitable as a result of the current acute illness, and either the treating clinician, the patient or the substitute decision maker are not committed to full active treatment; OR

14. Patients with a life expectancy < 30 days due to a chronic or underlying medical condition; OR

15. Considered to be at high risk of cerebral edema by the treating clinician (e.g. traumatic brain injury or acute brain disease); OR

16. Clinician believes that being enrolled in the intervention or control arm is not in the best interest of the patient; OR

17. Previous enrolment in this study.

Informed consent

The informed consent process was in accordance with legal requirements in each jurisdiction.

Intervention

Study drug preparation

Patients assigned to the Sodium Bicarbonate Group received a solution of sodium bicarbonate 8.4% (1,000mEq/L) diluted in a 500mL bag of 5% dextrose (300mL of 5% dextrose was removed and 300mL of sodium bicarbonate 8.4% was added). The final solution had 600mEq/L of sodium bicarbonate. Patients assigned to the Placebo Group received a standard 500mL 5% dextrose bag. Both study drugs were macroscopically identical, and the stability of the study drug and feasibility of blinding were validated in two previous studies.[2,3]

5% dextrose was chosen as the comparator because it is a readily available intravenous fluid that is used by clinicians and is a common diluent for drug administration. Moreover, 5% dextrose has an exemplary safety profile and does not lead to the hyperchloremia exacerbating metabolic acidosis seen with saline solutions. Importantly, balanced crystalloid solutions such as Compound Sodium Lactate were not suitable as a comparator because sodium bicarbonate is chemically incompatible with calcium-containing solutions. The presence of calcium can lead to precipitation of calcium carbonate when mixed with bicarbonate, posing risks of catheter occlusion and embolic complications, and precluding safe preparation, storage, and blinded administration of the study drug.

Intervention protocol

The intervention was informed by a survey circulated amongst healthcare professionals in the Australian and New Zealand Intensive Care Society Clinical Trials Group (ANZICS-CTG).[4] In both arms, a study drug infusion had to be started within one hour of randomization.

Both groups were treated using the same intervention protocol (Figure 1S). The study drug was continuously infused targeting a pH ≥ 7.30 and a base excess ≥ 0mEq/L. These targets reflect thresholds commonly used in clinical practice and research to define clinically significant acidosis based on evidence linking pH < 7.30 with impaired myocardial contractility, reduced catecholamine responsiveness, and increased risk of renal injury.[5,6] The base excess threshold provided an additional marker of metabolic correction beyond pH alone. The infusion was maintained until this target was achieved and continued by titration thereafter for a maximum of 5 hours (to maintain target pH and base excess levels). The infusion always started at 100mL/hour and was maintained at this rate until both targets were achieved, following which, the infusion rate was decreased to 25mL/h and kept constant at this rate until five hours had elapsed since the start of the infusion. At this point, the infusion was stopped, independently of the results of blood gas analysis. The titration strategy was designed to allow prompt correction of acidosis without overshooting the desired targets or exposing patients to excessive fluid or sodium loads. The algorithm used for titration is available in figure 1S, and an online decision support tool was available to all investigators to facilitate the titration process (available at https://bit.ly/soda-bic) (Figure 2S).

The study infusion was delivered for a maximum of five hours, until renal replacement therapy was started, the patient was discharged from the ICU, or the patient died, whichever occurred first. Blood gases were obtained at predefined timings within the first five hours (at 0, 1 [± 0.5], 3 [± 0.5], and 5 [± 1] hours after the start of the infusion). The study drug was permanently withheld if any contraindication to sodium bicarbonate developed (hypernatremia [Na > 155mEq/L], hypocalcemia [iCa < 0.8mmol/L], severe hypokalemia refractory to potassium replacement [K < 2.5mEq/L], clinically significant fluid overload [based on clinical assessment performed by the blinded bedside team], or any contraindication according to the treating physician).

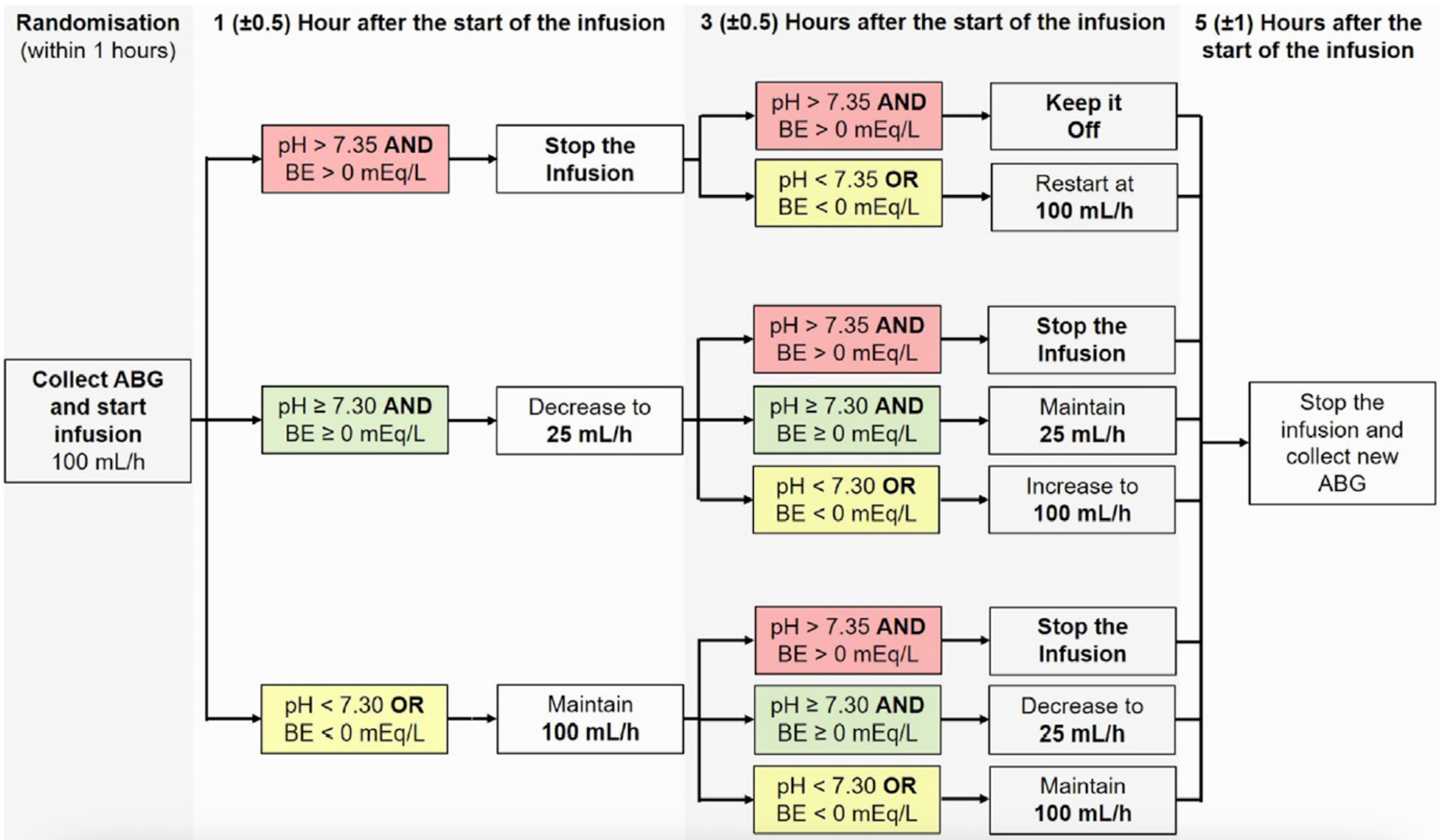

ABG - arterial blood gas; BE - base excess; SODa-BIC - Sodium Bicarbonate for Metabolic Acidosis in the ICU.

Figure 1S - Infusion and titration algorithm for patients included in SODa-BIC.

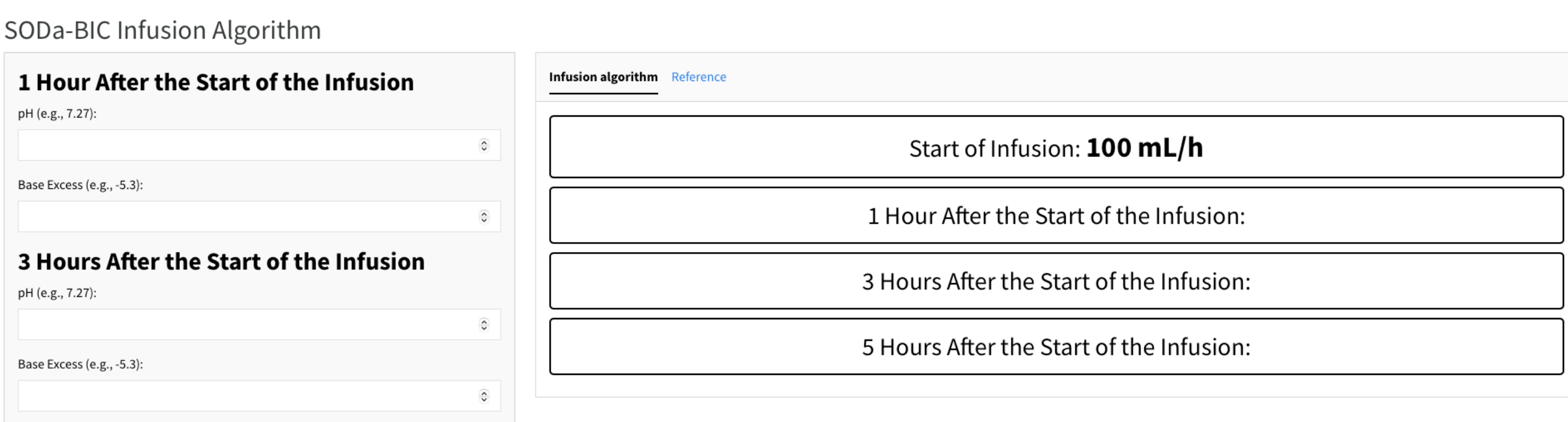

SODa-BIC - Sodium Bicarbonate for Metabolic Acidosis in the ICU.

Figure 2S - Online decision support tool for the study drug titration.

Available at https://bit.ly/soda-bic.

All other aspects of care were determined by the treating clinical team, including the use of additional fluid therapy, vasopressors, and other organ support modalities. The use of open-label sodium bicarbonate after trial inclusion was allowed but discouraged. However, circumstances might have arisen in which the use of open-label sodium bicarbonate was considered necessary by the treating clinicians. If this was the case, the following criteria were provided (but not mandatory) to ensure such administration was consistent with current practice:

1. pH < 7.10 in the presence of a $PaCO_2$ < 40mmHg.
2. Bicarbonate < 8mmol/L.
3. Base excess < -15mEq/L.
4. Severe hyperkalemia (K > 7.0mmol/L) in the presence of severe acidemia (pH < 7.10).

Data collection

Trained research staff collected data at each site, and these data were managed in a web-based case report form. Accuracy and consistency checks were carried out by way of automatic validation, and prespecified and *ad hoc* checking by personnel at the coordinating center. On-site data monitoring was performed by trained project managers and study monitors from the Australian and New Zealand Intensive Care Research Centre, supplemented by trained local contracted monitors where required, and with remote monitoring undertaken for selected sites.

After completion of data entry and resolution of data queries, the study database was reviewed and locked. The locked database was then exported for statistical analysis. Following database lock, investigator access was removed, and the database was archived.

OUTCOMES

Primary outcome

The primary outcome was major adverse kidney events within 30 days, a composite of death from any cause, receipt of renal replacement therapy, or persistent renal dysfunction (defined as a final inpatient creatinine value closest to day 30 ≥ 200% of the baseline value),[7,8] all censored at hospital discharge or 30 days, whichever occurred first (Table 1S). All patients who survived the index hospital admission were defined as alive at day 30. Major adverse kidney events within 30 days are a widely accepted outcome in clinical trials.[9-11] A value for baseline creatinine level was determined using a hierarchical approach in which the most recent creatinine value obtained within three months but more than seven days prior to the ICU admission was given priority over in-hospital measurements obtained less than seven days before ICU admission.[9-12] The baseline creatinine level was estimated with a previously described three-variable formula (creatinine = 0.74 – 0.2 [if female] + 0.08 [if African American] + 0.003 x age [in years]) when no pre-enrollment measurements were available.[12]

Secondary outcomes

Secondary outcomes were as follows: in-hospital death by day 30; receipt of renal replacement therapy within 30 days; persistent renal dysfunction within 30 days (defined as a final inpatient creatinine value closest to day 30 ≥ 200% of the baseline value); renal replacement therapy dependence at day 30 (defined by the receipt of any form of renal replacement therapy within ± 10 days of the 30-day time point following randomization); ICU mortality; and in-hospital death by day 90.

Physiological outcomes

Physiological outcomes were as follows: recurrence of metabolic acidosis (defined as pH < 7.30, base excess ≤ -4mEq/L and $PaCO_2$ ≤ 45mmHg; acidosis must have first resolved before a subsequent episode could be classified as a recurrence) in the first seven days after randomization; and incidence and maximum stage of acute kidney injury in the first seven days after randomization (based on creatinine criteria from Kidney Disease: Improving Global Outcomes criteria).[13]

Process of care measures

Process of care measures were as follows: vasopressor-free days at day 30 (defined as 30 – days receiving a continuous infusion of vasopressor [at any time and for any duration]; non-survivors at day 30 received 0); renal replacement therapy-free days at day 30 (defined as 30 – days receiving renal replacement therapy; non-survivors at day 30 received 0); ICU-free days at day 30 (defined as 30 – ICU length of stay; non-survivors at day 30 received 0); and hospital-free days at day 90 (defined as 90 – hospital length of stay; non-survivors at day 90 received 0).

Table 1S - Major adverse kidney events within 30 days definition

Component	Definition	Sensitivity analysis
Death from any cause within 30 days	Any death occurred between randomization, hospital discharge, or day 30 after randomization, whichever comes first	Any death occurred between randomization, hospital discharge, or day 30 after randomization, whichever comes first
Receipt of renal replacement therapy within 30 days	Any receipt of renal replacement therapy between randomization, hospital discharge, or day 30 after randomization, whichever comes first	Any receipt of renal replacement therapy between randomization, hospital discharge, or day 30 after randomization, whichever comes first
Persistent renal dysfunction at day 30	Final inpatient creatinine value closest to day 30 ≥ 200% of the baseline value	Final inpatient eGFR value closest to day 30 < 75% of estimated baseline value (i.e. a > 25% decrease in eGFR)

eGFR - estimated glomerular filtration rate.

Prespecified subgroup analysis

Treatment effects on incidence of major adverse kidney events within 30 days was analyzed according to the following predefined subgroups: 1) pH level (< 7.25 *versus* ≥ 7.25); 2) acute kidney injury (stage 2 or 3 *versus* none or stage 1 according to the Kidney Disease: Improving Global Outcomes criteria[13]); 3) $PaCO_2$ level (45-50 *versus* ≤ 45mmHg); 4) type of acidosis (high anion gap [> 12mEq/L] *versus* non-anion gap [≤ 12 mEq/L]); 5) severity of illness (> median *versus* ≤ median of APACHE II); 6) received bicarbonate pre-randomization (yes *versus* no); 7) septic shock (yes *versus* no); and 8) type of patient (medical *versus* surgical). Analyses of heterogeneity of effects across subgroups were performed with the use of a treatment-by-subgroup interaction term added to the primary model and were presented in a forest plot. Sepsis was defined as suspected or confirmed infection together with a total (or modified) SOFA score ≥ 2. Septic shock was defined as sepsis requiring vasopressor support and a lactate concentration > 2mmol/L.

STATISTICAL ANALYSIS

Sample size calculation and re-estimation

Assuming a control group major adverse kidney events within 30 days rate of 40% (a conservative approach based on published studies and data from the Australian and New Zealand Intensive Care Society Centre for Outcome and Resource Evaluation Adult Patient Database),[6,14] a sample size of 470 provides 90% power (2-sided α of 0.05) to detect an absolute difference of 14%. This estimate was informed by the BICAR-ICU trial,[15] which reported a 10% absolute reduction in 30-day mortality, a 17% reduction in renal replacement therapy, and a 21% reduction in the composite of mortality and/or renal replacement therapy in selected subgroups of critically ill patients with severe metabolic acidosis. Given that SODa-BIC included a broader ICU population, including patients with less severe acidosis, we anticipated a more modest treatment effect. Therefore, we conservatively assumed 70% of the treatment effect reported in the BICAR-ICU trial. This adjustment accounts for expected attenuation of benefit in a more heterogeneous population while still allowing detection of a clinically meaningful difference. To account for potential loss to follow-up or withdrawal of consent, the final sample size was increased to 500 patients.

SODa-BIC employed an adaptive design considering a blinded sample size re-estimation after the collection of outcomes for 100 patients. An independent statistician who remained unaware of treatment allocation estimated the aggregated

incidence of the primary outcome across both study groups. Using this observed event rate and assuming the originally specified relative risk reduction, the required total sample size was recalculated to maintain adequate statistical power. To ensure both feasibility and protection against potential under-powering, predefined boundaries were applied to the re-estimated sample size. If the recalculated sample size exceeded 700 participants, recruitment would be capped at this level. Conversely, if the observed event rate was higher than initially assumed, the total sample size would not be reduced below the originally planned 500 participants. This conservative approach was intended to preserve statistical power in the presence of uncertainty regarding baseline risk while avoiding premature reduction in sample size. The results of the blinded sample size re-estimation were reviewed by the independent DSMC, which recommended continuation of the trial with the originally planned sample size.

Confidence distribution

To support interpretation of the treatment effect, we calculated a confidence distribution for the estimated absolute risk difference in the primary outcome using a normal approximation based on the point estimate and its standard error derived from the prespecified mixed-effects model (including treatment group and stratification factors as fixed effects and sites as a random effect).[16] The confidence distribution offers a full frequentist representation of uncertainty around the estimated treatment effect and allows calculation of the confidence that the absolute risk difference is less than (or greater than) a clinically meaningful threshold (e.g., 0). This approach is analogous to calculating the Bayesian posterior probability of benefit but does not require a prior distribution. This method improves interpretability for clinicians and decision-makers by directly quantifying the confidence that the intervention is beneficial (i.e., that the absolute difference is < 0). The confidence distribution was computed to provide the frequentist probability that the absolute difference was less than 0.[16,17]

Prespecified analyses not reported in the present manuscript

The trial protocol included additional prespecified analyses that are not reported in the present manuscript, including planned health economic and longer-term outcome analyses. These analyses will be reported separately.

Adverse events

1. Consistent with established practice in ICU trials,[18] events that are part of the natural history of the primary disease process or expected complications of critical illness were not reported as serious adverse events in this study. All adverse events considered to be potentially causally related to the study intervention or otherwise of concern in the investigator's judgment were reported. In addition, the following adverse events were recorded only during study drug infusion:
2. Hypokalemia (K < 3.0mmol/L) needing correction;
3. Hypocalcemia (iCa < 0.8mmol/L) needing correction; and
4. Dysnatremia (Na > 155mEq/L);
5. Hypercapnia ($PaCO_2$ > 50mmHg);

Hypervolemia (PaO_2/FiO_2 < 100 due to hypervolemia [not other cause] and clinician concern).

Case report form

All study data were collected from the patient's medical record by research staff at each participating site and entered into a secure electronic Case Report Form (eCRF).

Source data verification and monitoring

Source data verification and monitoring activities were performed at each site by a study monitor or the study Project Manager. On-site data monitoring was performed by trained project managers and study monitors from the Australian and New Zealand Intensive Care Research Centre, supplemented by trained local contracted monitors where required, and with remote monitoring undertaken for selected sites. 100% source data verification was performed on all key variables for

all patients. Key variables refer to trial data that are of high importance to the safety, well-being, and rights of patients, as well as the achievement of the trial objectives. 100% source data verification was also completed for the first two patients at all sites.

Protocol changes

Amendments to the original protocol are as follows.

Protocol version	Protocol date	Summary of changes
1.1	16 May 2023	Administrative changes, addition of guidelines around open-label sodium bicarbonate use, clarification of inclusion and exclusion criteria
1.2	8 November 2023	Addition of follow-up at 180 days
2.0	8 April 2024	Change of inclusion criteria expanding the $PaCO_2$ criteria (increase of $PaCO_2$ threshold from 45 to 50mmHg in intubated and ventilated patients)

Co-enrolment

Co-enrolment in the SODa-BIC study and other clinical trials was encouraged but was subject to a formal review process. The SODa-BIC study Management Committee and the relevant Management Committee for other clinical trials reviewed each other's study protocols, and if both Committees agreed that there was no scientific, ethical or procedural impediment to co-enrolment, then co-enrolment was allowed. A co-enrolment table for the SODa-BIC study was kept up-to-date and available on the study database. The following clinical trials were approved for co-enrolment by the SODa-BIC study Management Committee:

Study name	Full study name	Trial Registration No.
AQUA	Assessment of quantity and utility of water for ICU-acquired hypernatraemia. a pilot, six-centre, open-label, safety and physiological efficacy randomized controlled trial	ANZCTR ACTRN12625000904471
ARISE-FLUIDS	Australasian resuscitation in sepsis evaluation: fluid or vasopressors in emergency department sepsis	ClinicalTrials.gov NCT04569942
BONE-ZONE	Bone loss prevention with zoledronic acid or denosumab in critically ill women – a randomised controlled trial	ClinicalTrials.gov NCT04608630
CALIPSO	Duration of cardiac antimicrobial prophylaxis outcomes	ClinicalTrials.gov NCT05447559
CLIP-II	The cryopreserved *versus* liquid platelets trial	ClinicalTrials.gov NCT03991481
CONTINUE	Continuation of Enteral nutrition prior to extubation compared to standard care (CONTINUE): a pilot randomised controlled trial	ClinicalTrials.gov NCT06382727
DECIDE	Dexmedetomidine in cardiac surgical intraoperative drug evaluation	ANZCTR ACTRN12623001171606
DEPOSITION	Decreasing postoperative blood loss by topical *versus* intravenous tranexamic acid in open cardiac surgery	ClinicalTrials.gov NCT03954314
DIRECT	Guiding energy provision using indirect calorimetry: a pilot feasibility randomised controlled trial in critically ill adults with obesity	ClinicalTrials.gov NCT06053216.
DRIVE	DRIVing PrEssure limited ventilation in hypoxemic respiratory failure–randomised control trial	ClinicalTrials.gov NCT05440851
ECMO-PROMPT	A pilot feasibility RCT of patient-reported outcome measure monitoring and feedback to improve disability and quality of life in critically ill patients who survive extracorporeal membrane oxygenation	ANZCTR ACTRN12624000293561
EPO-TRAUMA	A randomised controlled trial of erythropoietin in trauma	ANZCTR ACTRN12619001632189P
HOME-AKI	Haemofiltration with Oxiris: Multicentre Evaluation in Acute Kidney Injury (HOME-AKI). A pilot study comparing two devices used for renal replacement therapy in the intensive care unit in patients with acute kidney injury related to sepsis.	ANZCTR ACTRN12620000514909p
Inspire Beyond ICU	Effect of inspiratory muscle training on quality of life in ICU patients requiring mechanical ventilation: a multicentre randomised trial	ANZCTR ACTRN12625000027415

ITACS	IV iron for treatment of anaemia before cardiac surgery	ClinicalTrials.gov NCT02632760
iTHRIVE	Iron and erythropoietin use in anaemic patients in the intensive care	ANZCTR ACTRN12621000595819
MEGA-ROX	The mega randomised registry trial comparing conservative *versus* liberal oxygenation targets	ANZCTR ACTRN12620000391976
NOTACS	Effect of nasal high-flow oxygen therapy on patient-centred outcomes in patients at high risk of postoperative pulmonary complications after cardiac surgery: a multicentre randomised controlled trial	ISRCTN14092678
PORTHOS	Angiotensin II *versus* noradrenaline infusion to reduce length of hospital stay after cardiac surgery	ANZCTR ACTRN12623000848606
PREVENT	A randomized, sham controlled, double-blinded, multi-center trial to evaluate the efficacy of the ventfree respiratory muscle stimulator to assist ventilator weaning in critically ill patients	ANZCTR ACTRN12625000904471
PREVENTS-AKI	PREVENTion with SGLT-2 inhibition of acute kidney injury in intensive care	ClinicalTrials.gov NCT05468203
PRICE-1	Liberal versus restrictive phosphate replacement for critically ill patients treated in an intensive care unit: a pilot, cluster, crossover, randomised, controlled trial	ANZCTR ACTRN12622001193763p
REACT-SHOCK	Relative hypotension and acute kidney injury in patients with shock: a prospective multicentre cohort study	ANZCTR ACTRN12623000044628
REMAP-CAP	A randomised, embedded, multi-factorial, adaptive platform trial for community-acquired pneumonia	ClinicalTrials.gov NCT02735707
REVISE	Re-EValuating the Inhibition of Stress Erosions and prophylaxis against gastrointestinal bleeding in the critically ill	ClinicalTrials.gov NCT03374800
ROSETTA	Red blood cell transfusion in ECMO – a feasibility trial	ClinicalTrials.gov NCT05814094
SNAP	Staphylococcus aureus network adaptive platform trial	ClinicalTrials.gov NCT05137119
SPICE IV	Early sedation with dexmedetomidine *versus* placebo in older ventilated	ANZCTR ACTRN12620001088932p
STEPCARE	Sedation, temperature and pressure after cardiac arrest and resuscitation – The STEPCARE trial	ClinicalTrials.gov NCT05564754
T4P	The threshold for platelets study: a prospective randomised trial to define the platelet count below which critically ill patients should receive a platelet transfusion prior to an invasive procedure	ISRCTN79371664
TRITON	A Feasibility Trial of TRanexamic AcId for NecroTising SOft-tissue INfections	ANZCTR ACTRN12621001423808

Table 2S - Characteristics of the intervention in the first 24 hours*

	Sodium bicarbonate (n = 244)	Placebo (n = 254)	Absolute difference (95%CI)
Median hours between randomization and start of infusion	0.52 (0.39 - 0.72)	0.38 (0.27 - 0.53)	0.14 (0.09 - 0.19)
Median duration of infusion (hours)	5.0 (4.7 - 5.0)	5.0 (5.0 - 5.0)	-0.0 (-0.0 - 0.0)
Median volume infused (mL)	500 (329 - 500)	500 (500 - 500)	-0.0 (-32.0 - 32.0)
Median total sodium bicarbonate dose (mmol)	300.0 (197.4 - 300.0)	0.0 (0.0 - 0.0)	300.0 (292.4 - 307.6)
Titration of study drug infusion†	121 / 243 (49.8)	58 / 250 (23.2)	27.0 (19.5 - 34.5)
Use of open-label sodium bicarbonate	17 / 243 (7.0)	38 / 250 (15.2)	-8.2 (-13.6 - -2.8)
Blood gas analysis during the first 24 hours*			
Median pH			
Lowest	7.31 (7.26 - 7.34)	7.26 (7.22 - 7.30)	0.05 (0.04 - 0.06)
Highest	7.43 (7.39 - 7.46)	7.37 (7.33 - 7.41)	0.05 (0.04 - 0.06)
Median bicarbonate (mmol/L)			
Lowest	19.0 (17.0 - 22.0)	17.0 (15.0 - 19.0)	2.6 (1.7 - 3.4)
Highest	26.0 (23.8 - 27.9)	21.0 (19.0 - 23.0)	4.5 (3.8 - 5.2)
Median base excess (mmol/L)			
Lowest	-6.0 (-9.0 - -3.5)	-9.0 (-12.0 - -7.0)	3.1 (2.4 - 3.7)
Highest	1.5 (-1.0 - 3.0)	-4.2 (-6.2 - -1.8)	5.1 (4.4 - 5.8)
Median $PaCO_2$ (mmHg)			
Lowest	35.0 (32.0 - 38.9)	33.9 (30.0 - 37.0)	1.0 (-0.2 - 2.2)
Highest	44.0 (40.0 - 48.0)	41.8 (38.0 - 46.0)	2.0 (0.6 - 3.4)
Median lactate (mmol/L)			
Lowest	1.3 (1.0 - 2.2)	1.3 (0.9 - 1.9)	0.1 (-0.0 - 0.2)
Highest	3.1 (2.1 - 5.4)	2.9 (1.8 - 5.1)	0.2 (-0.1 - 0.5)
Median sodium (mEq/L)			
Lowest	136.5 (134.0 - 140.0)	133.0 (131.0 - 136.0)	4.0 (2.6 - 5.4)
Highest	141.0 (138.0 - 144.0)	137.0 (134.0 - 140.0)	4.0 (2.6 - 5.4)
Median noradrenaline infusion rate (μg/min)			
Lowest	0.06 (0.03 - 0.10)	0.05 (0.02 - 0.12)	0.00 (-0.01 - 0.01)
Highest	0.18 (0.10 - 0.31)	0.19 (0.11 - 0.30)	-0.01 (-0.04 - 0.02)

* Variable derived from measurements obtained at six time points during the first 24 hours after randomization and initiation of the study drug infusion (1, 3, 5, 12, 18, and 24 hours). The study drug was infused until hour 5; †titration was defined as any protocol-driven change in infusion rate from the initial 100mL/hour during the 5-hour study infusion, based on predefined assessments at one and three hours after the start of the infusion. Data are median (interquartile range), n/total (%) or n (%).

Table 3S - Trial outcomes

	Sodium bicarbonate (n = 244)	Placebo (n = 254)	Absolute difference (95% CI)	p value	Hazard ratio (95%CI)
Major adverse kidney events within 30 days	98 (40.2)	100 (39.4)	1.2 (-7.1 - 9.4)	0.78	
Secondary outcomes – components of the primary outcome*					
In-hospital death by day 30	62 (25.4)	61 (24.0)	1.8 (-5.6 - 9.2)		1.09 (0.76 - 1.56)
Receipt of renal replacement therapy within 30 days	41 (16.8)	53 (20.9)	-3.9 (-10.6 - 2.7)		
Persistent renal dysfunction within 30 days†	34 / 243 (14.0)	46 / 251 (18.3)	-4.3 (-10.7 - 2.1)		
Other secondary outcomes					
Renal replacement therapy dependence at day 30‡	14 (5.7)	23 (9.1)	-3.3 (-7.8 - 1.3)		
ICU mortality	49 (20.1)	56 (22.0)	-1.7 (-8.6 - 5.3)		
In-hospital death by day 90	64 (26.2)	65 (25.6)	1.2 (-6.3 - 8.6)		1.06 (0.75 - 1.51)
Physiological outcomes					
Recurrence of metabolic acidosis§	78 (32.0)	141 / 253 (55.7)	-23.3 (-31.4 - -15.1)		
Acute kidney injury¶	156 / 242 (64.5)	162 / 250 (64.8)	-0.0 (-7.7 - 7.6)		
Stage 1	64 / 156 (41.0)	58 / 162 (35.8)			
Stage 2	35 / 156 (22.4)	44 / 162 (27.2)			
Stage 3	57 / 156 (36.5)	60 / 162 (37.0)			
Process of care measures					
Median vasopressor-free days at day 30||	27.2 (0.0 - 28.9)	27.6 (6.1 - 28.9)	-0.5 (-1.3 - 0.4)		
Median renal replacement therapy-free days at day 30||	30.0 (0.0 - 30.0)	30.0 (10.4 - 30.0)	-0.0 (-0.6 - 0.6)		
Median ICU-free days at day 30||	23.7 (0.0 - 26.9)	24.0 (0.0 - 27.0)	-0.5 (-2.3 - 1.3)		
Median hospital-free days at day 90||	69.3 (0.0 - 79.8)	69.2 (0.0 - 80.6)	1.1 (-6.4 - 8.6)		

ICU - intensive care unit. * Components of the composite outcome were not mutually exclusive; some participants contributed to more than one component; † defined as a final inpatient creatinine value closest to day 30 ≥ 200% of the baseline value; ‡ defined by the receipt of any form of renal replacement therapy within ± 10 days of the 30-day time point following randomization; § defined as meeting criteria for metabolic acidosis (pH < 7.30, base excess ≤ -4mEq/L, and $PaCO_2$ ≤ 45mmHg) during the first seven days after randomization, including during the study infusion period. Acidosis must have first resolved before a subsequent episode could be classified as a recurrence; ¶ based on the creatinine criteria from KDIGO definition and in the first seven days after randomization. When not available, premorbid creatinine was imputed using the following formula: creatinine = 0.74 − 0.2 [if female] + 0.08 [if African American] + 0.003 × age [in years]; || defined as 30 / 90 – days receiving a continuous infusion of vasopressor / renal replacement therapy / in ICU / in hospital; non-survivors at day 30 / 90 received 0. Data are median (interquartile range), n/total (%) or n (%). The widths of the confidence intervals for secondary outcomes have not been adjusted for multiple comparisons, so the intervals should not be used to infer definitive differences between groups.

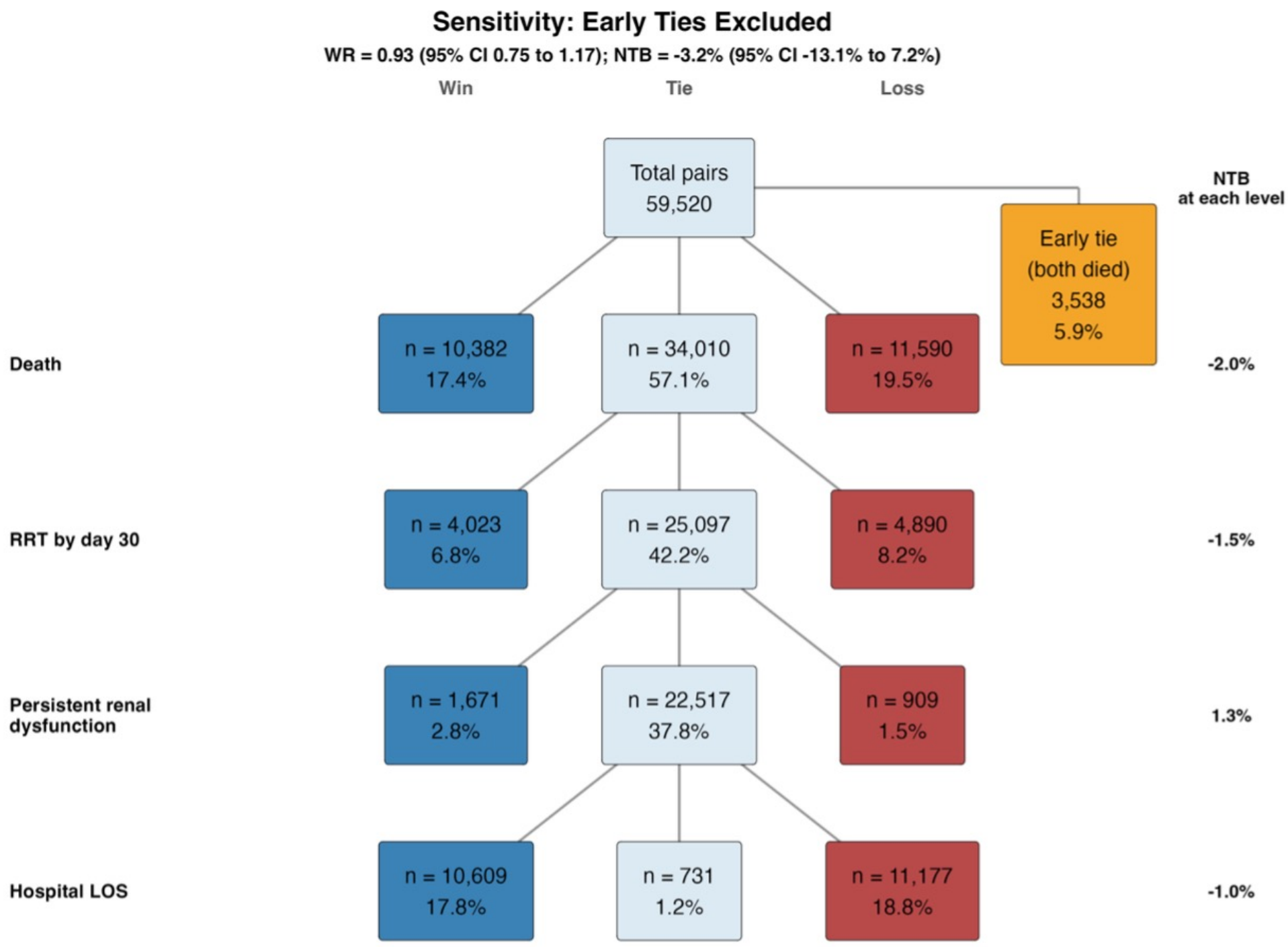

WR - win ratio; NTB - net treatment benefit, calculated as (wins — losses) / total number of pairwise comparisons; RRT - renal replacement therapy; LOS - length of stay.

Figure 3S - Win ratio hierarchy analysis of the primary outcome excluding early ties.

Distribution of wins, losses, and ties across the hierarchical components of the primary Win Ratio analysis. Outcomes were evaluated sequentially according to the predefined hierarchy of death by day 30, receipt of renal replacement therapy by day 30, persistent renal dysfunction by day 30, and hospital length of stay. Comparisons not resolved at a higher level progressed to the next level of the hierarchy. Percentages represent the proportion of all pairwise comparisons resolved at each level of the hierarchy. Early ties occurring after death comparisons were excluded (if both patients in the pair died, they were excluded from the analyses at this point, and no further comparisons were made at lower levels of the hierarchy).

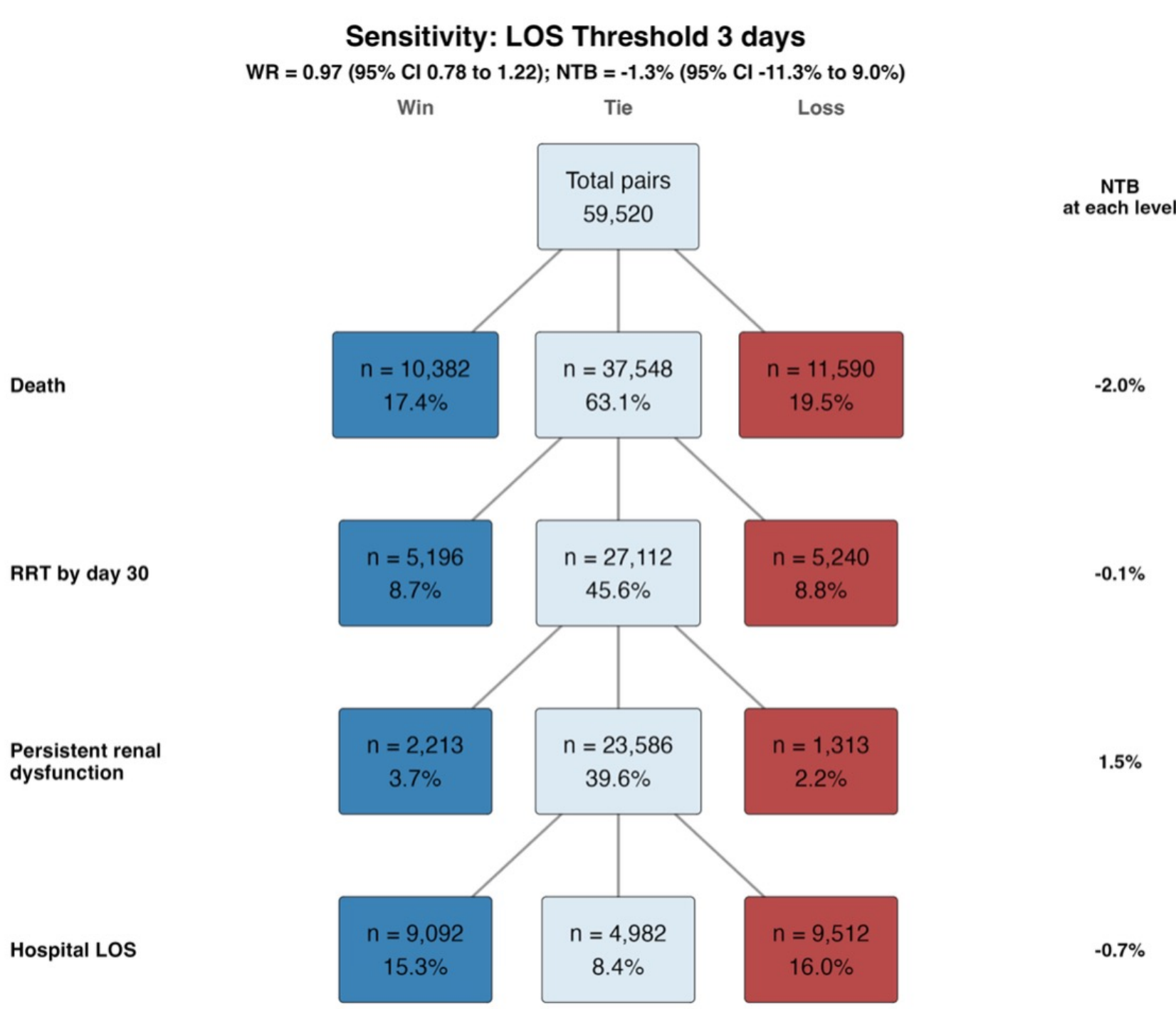

WR - win ratio; NTB - net treatment benefit, calculated as (wins — losses) / total number of pairwise comparisons; RRT - renal replacement therapy; LOS - length of stay;

Figure 4S - Win ratio hierarchy analysis of the primary outcome including a different threshold for hospital length of stay.

Distribution of wins, losses, and ties across the hierarchical components of the primary win ratio analysis. Each patient assigned to sodium bicarbonate was compared with every patient assigned to placebo, generating 59,520 pairwise comparisons. Outcomes were evaluated sequentially according to the predefined hierarchy of death by day 30, receipt of renal replacement therapy by day 30, persistent renal dysfunction by day 30, and hospital length of stay. Comparisons not resolved at a higher level progressed to the next level of the hierarchy. Percentages represent the proportion of all pairwise comparisons resolved at each level of the hierarchy. Application of a minimum three-day difference in hospital length of stay to define a win (if a pair was being compared at the length of stay level, then to record a win, the length of stay had to be at least three days shorter than the comparison).

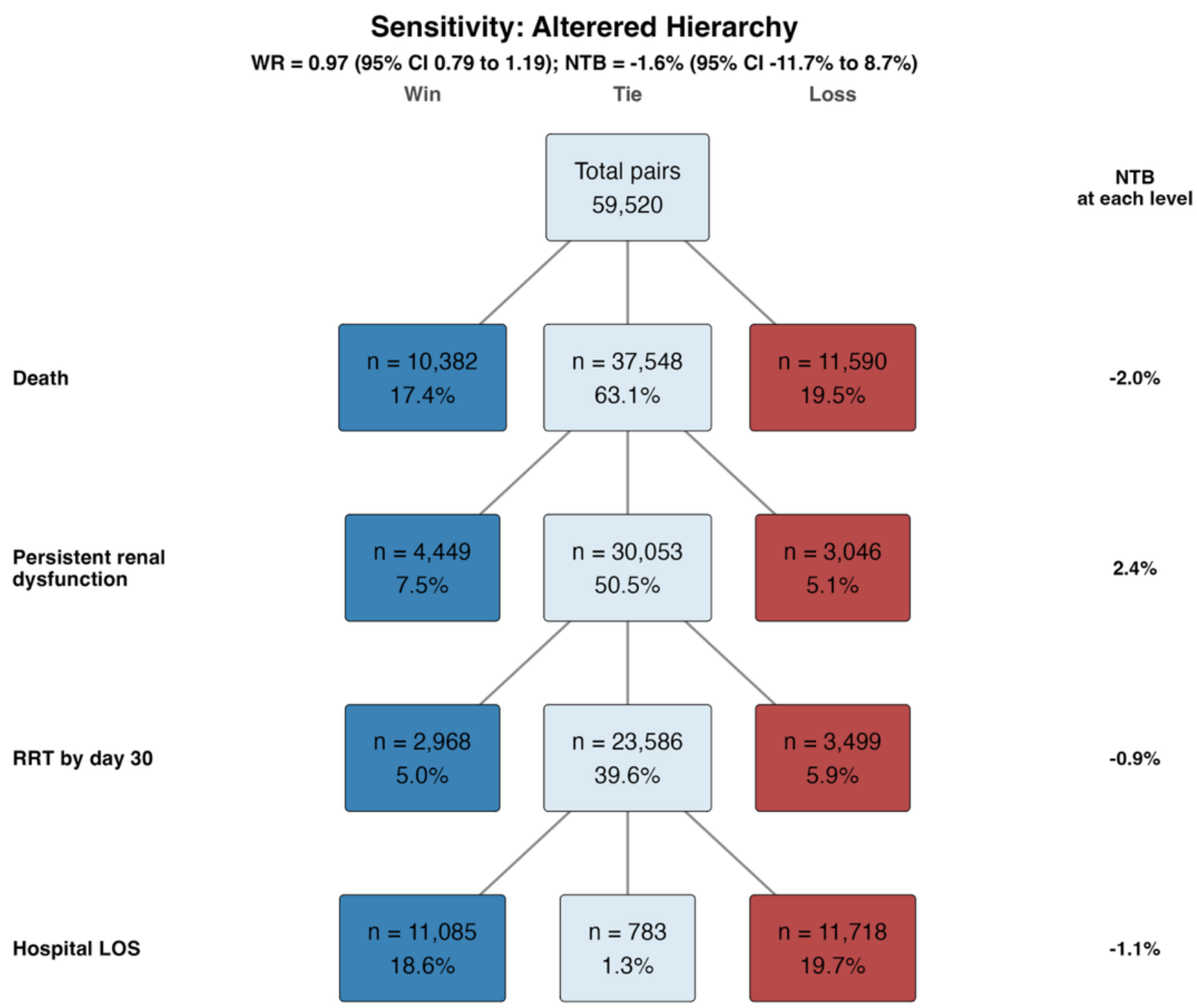

WR - win ratio; NTB - net treatment benefit, calculated as (wins — losses) / total number of pairwise comparisons; RRT - renal replacement therapy; LOS - length of stay.

Figure 5S - Win ratio hierarchy analysis of the primary outcome including an alternate hierarchy.

Distribution of wins, losses, and ties across the hierarchical components of the primary win ratio analysis. Each patient assigned to sodium bicarbonate was compared with every patient assigned to placebo, generating 59,520 pairwise comparisons. Outcomes were evaluated sequentially according to the predefined hierarchy of death by day 30, receipt of renal replacement therapy by day 30, persistent renal dysfunction by day 30, and hospital length of stay. Comparisons not resolved at a higher level progressed to the next level of the hierarchy. Percentages represent the proportion of all pairwise comparisons resolved at each level of the hierarchy. Application of an alternate hierarchy whereby persistent renal dysfunction was assessed before renal replacement therapy.

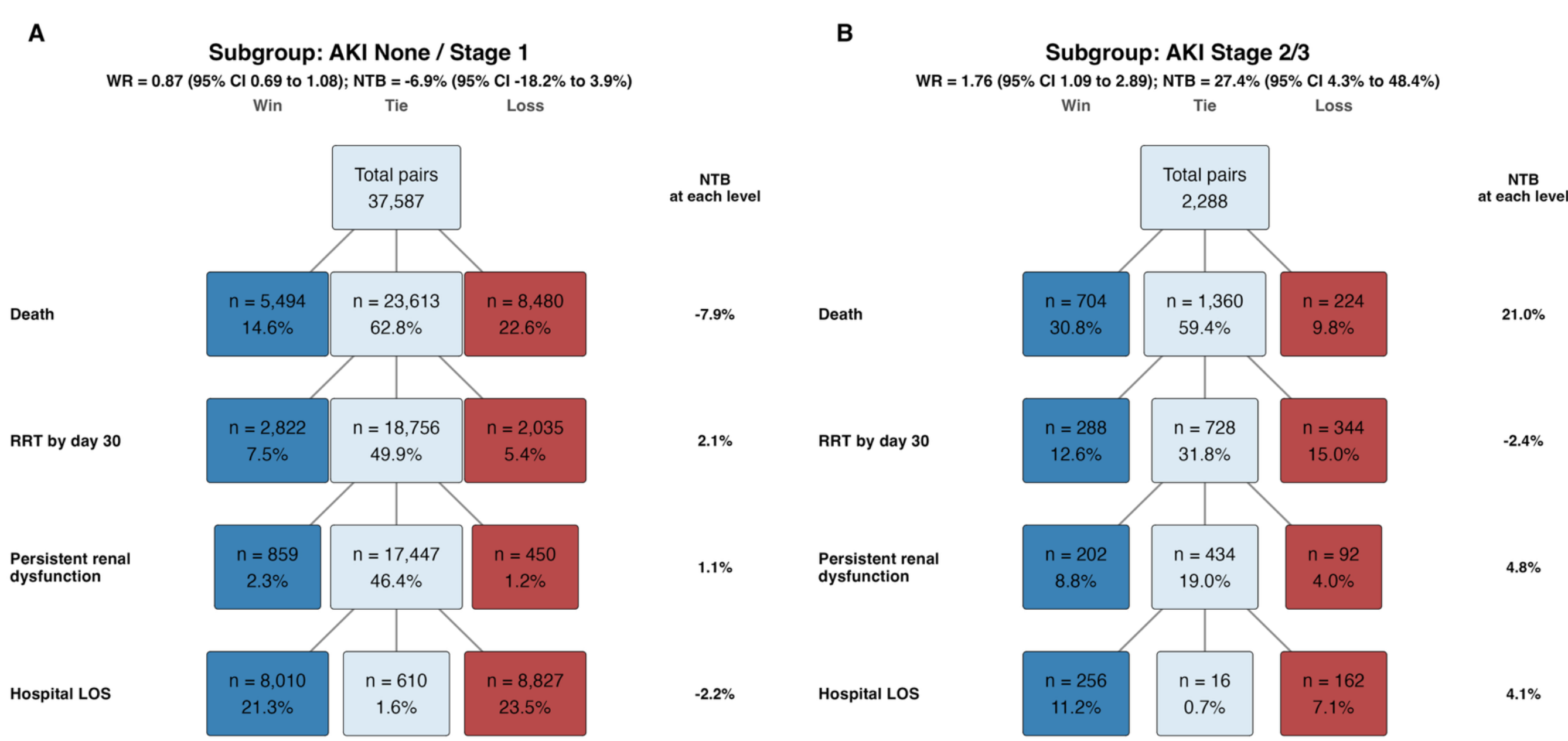

WR - win ratio; NTB - net treatment benefit, calculated as (wins — losses) / total number of pairwise comparisons; RRT - renal replacement therapy; LOS - length of stay;

Figure 6S - Subgroup analyses of the win ratio for patients with and without acute kidney injury.

Distribution of wins, losses, and ties across the hierarchical components of the primary win ratio analysis. Each patient assigned to sodium bicarbonate was compared with every patient assigned to placebo. Outcomes were evaluated sequentially according to the predefined hierarchy of death by day 30, receipt of renal replacement therapy by day 30, persistent renal dysfunction by day 30, and hospital length of stay. Comparisons not resolved at a higher level progressed to the next level of the hierarchy. Percentages represent the proportion of all pairwise comparisons resolved at each level of the hierarchy. Acute kidney injury based on the creatinine criteria from Kidney Disease: Improving Global Outcomes (KDIGO) definition.

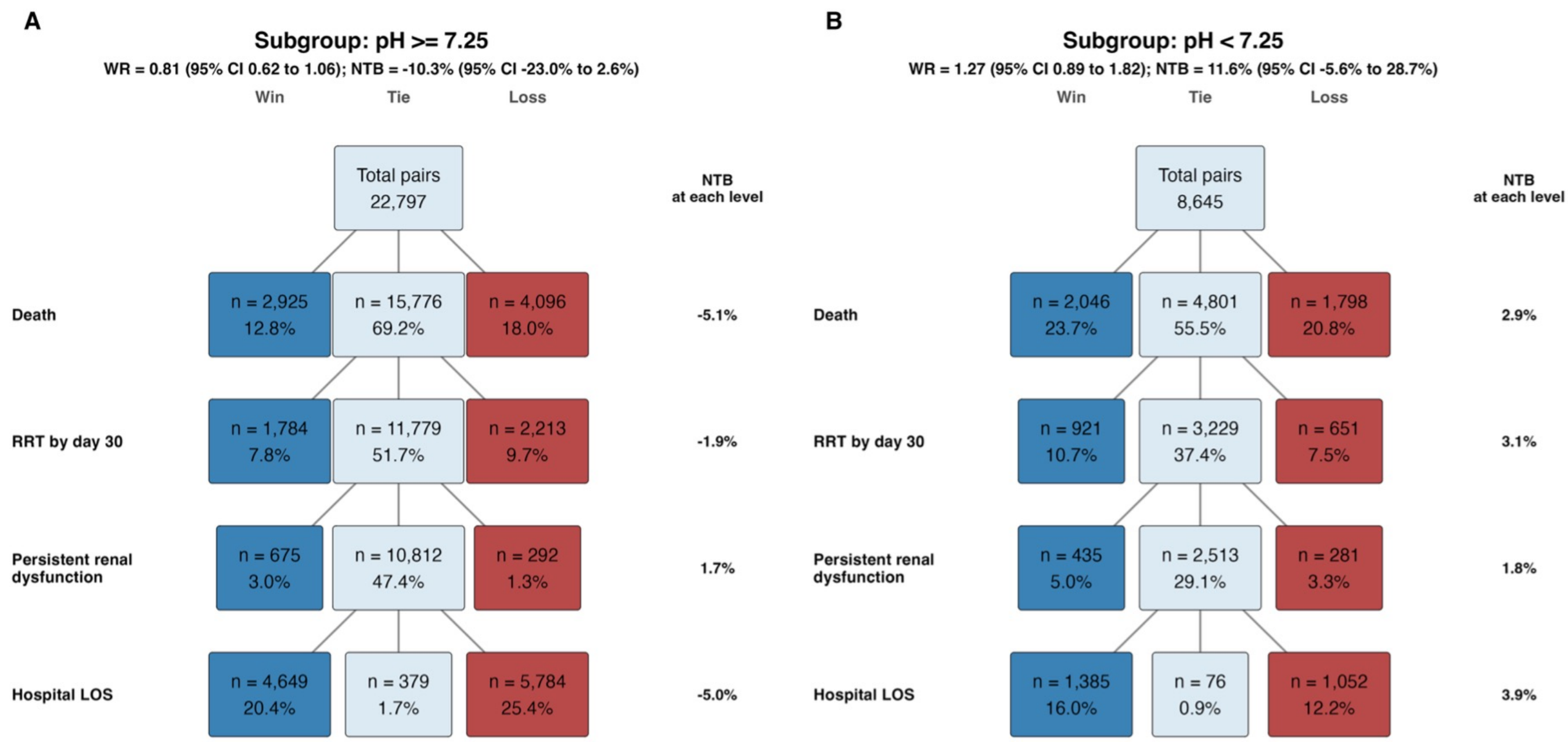

WR - win ratio; NTB is net treatment benefit, calculated as (wins — losses) / total number of pairwise comparisons; at each level of the hierarchy. RRT - renal replacement therapy; LOS is length of stay.

Figure 7S - Subgroup analyses of the win ratio according to the severity of acidemia.

Distribution of wins, losses, and ties across the hierarchical components of the primary win ratio analysis. Each patient assigned to sodium bicarbonate was compared with every patient assigned to placebo. Outcomes were evaluated sequentially according to the predefined hierarchy of death by day 30, receipt of renal replacement therapy by day 30, persistent renal dysfunction by day 30, and hospital length of stay. Comparisons not resolved at a higher level progressed to the next level of the hierarchy. Percentages represent the proportion of all pairwise comparisons resolved.

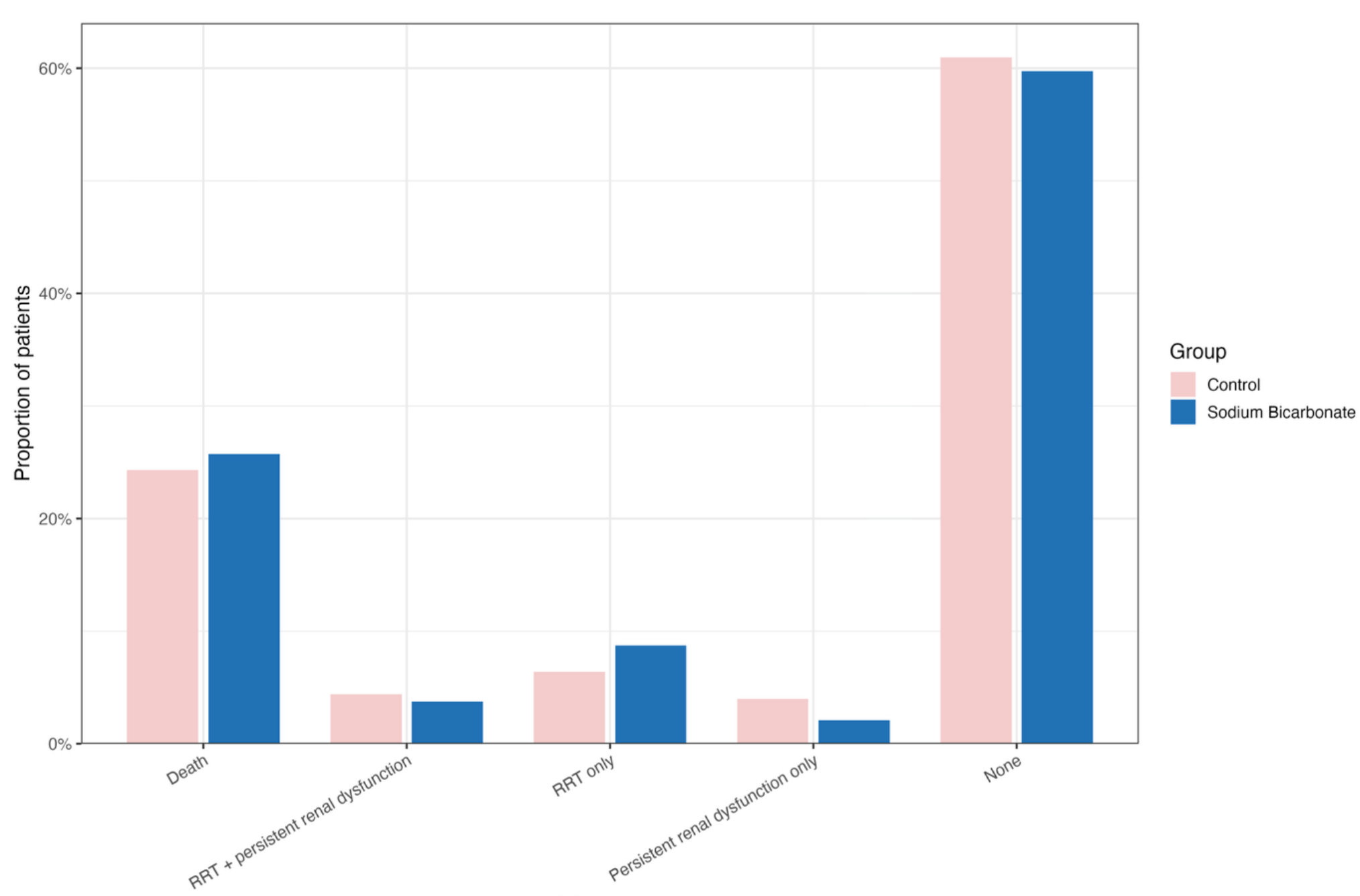

RRT - renal replacement therapy; DOOR - Desirability of Outcome Ranking.

Figure 8S - Distribution of Desirability of Outcome Ranking (DOOR) categories according to treatment allocation.

Distribution of participants across the five mutually exclusive DOOR categories according to treatment allocation. Categories were ranked from least to most desirable outcome as follows: 1) in-hospital death by day 30; 2) in-hospital survival at day 30 with both renal replacement therapy and persistent renal dysfunction; 3) in-hospital survival at day 30 with renal replacement therapy but without persistent renal dysfunction; 4) in-hospital survival at day 30 with persistent renal dysfunction but without renal replacement therapy; and 5) in-hospital survival at day 30 without renal replacement therapy or persistent renal dysfunction. The Better DOOR Probability was 0.49 (95%CI 0.45 - 0.54), indicating no overall difference between sodium bicarbonate and placebo. Bars represent the proportion of participants within each treatment group classified into each DOOR category. The DOOR analysis adjusted for Hospital length of stay, incorporating hospital length of stay as a tie-breaker, yielded a Better DOOR Probability of 0.49 (95%CI 0.43 - 0.54). A Better DOOR Probability greater than 0.50 favors sodium bicarbonate, whereas a Better DOOR Probability less than 0.50 favors placebo.

REFERENCES

1. Serpa Neto A, McNamara M, Cooper J, Fujii T, Higgins A, Hodgson C, et al.; SODa-BIC investigators. Protocol summary and statistical analysis plan for the sodium bicarbonate for metabolic acidosis in the intensive care unit (SODa-BIC) trial. Crit Care Resusc. 2025;27(2):100108.
2. Jude B, Naorungroj T, Neto AS, Fujii T, Udy A, Bellomo R. Sodium bicarbonate in 5% dextrose: can clinicians tell the difference? Crit Care Resusc. 2020;22(1):80-2.
3. Naorungroj T, Neto AS, Fujii T, Jude B, Udy A, Bellomo R. Stability of bicarbonate in normal saline: a technical report. Crit Care Resusc. 2020;22(1):83-5.
4. Serpa Neto A, Fujii T, El-Khawas K, Udy A, Bellomo R. Sodium bicarbonate therapy for metabolic acidosis in critically ill patients: a survey of Australian and New Zealand intensive care clinicians. Crit Care Resusc. 2020;22(3):275-80.
5. Jung B, Rimmele T, Le Goff C, Chanques G, Corne P, Jonquet O, et al.; AzuRea Group. Severe metabolic or mixed acidemia on intensive care unit admission: incidence, prognosis and administration of buffer therapy. A prospective, multiple-center study. Crit Care. 2011;15(5):R238.
6. Mochizuki K, Fujii T, Paul E, Anstey M, Pilcher DV, Bellomo R. Early metabolic acidosis in critically ill patients: a binational multicentre study. Crit Care Resusc. 2023;23(1):67-75.
7. Palevsky PM, Molitoris BA, Okusa MD, Levin A, Waikar SS, Wald R, et al. Design of clinical trials in acute kidney injury: report from an NIDDK workshop on trial methodology. Clin J Am Soc Nephrol. 2012;7(5):844-50.
8. Kellum JA, Zarbock A, Nadim MK. What endpoints should be used for clinical studies in acute kidney injury? Intensive Care Med. 2017;43(6):901-3.
9. Weisbord SD, Gallagher M, Jneid M, Garcia S, Cass A, Thwin S, et al; PRESERVE Trial Group. Outcomes after angiography with sodium bicarbonate and acetylcysteine. N Engl J Med 2018;378(7):603-14.
10. Semler MW, Self WH, Wanderer JP, Ehrenfeld JM, Wang L, Byrne DW, et al; SMART Investigators and the Pragmatic Critical Care Research Group. Balanced crystalloids versus saline in critically ill adults. N Engl J Med. 2018;378(9):829-39.
11. Self WH, Semler MW, Wanderer JP, Wang L, Byrne DW, Collins SP, et al; SALT-ED Investigators. Balanced crystalloids versus saline in noncritically ill adults. N Engl J Med. 2018;378(9):819-28.
12. Zavada J, Hoste E, Cartin-Ceba R, Calzavacca P, Gajic O, Clermon G, et al; AKI6 investigators. A comparison of three methods to estimate baseline creatinine for RIFLE classification. Nephrol Dial Transplant. 2010;25(12):3911-8.
13. Kidney Disease: Improving Global Outcomes (KDIGO) CKD Work Group. KDIGO 2024 Clinical Practice Guideline for the Evaluation and Management of Chronic Kidney Disease. Kidney Int. 2024;105(4S):S117-S314.
14. Fujii T, Udy AA, Nichol A, Bellomo R, Deane AM, El-Khawas K, et al.; SODA-BIC investigators. Incidence and management of metabolic acidosis with sodium bicarbonate in the ICU: an international observational study. Crit Care. 2021;25(1):45.
15. Jaber S, Paugam C, Futier E, Lefrant J, Lasocki S, Lescot T, et al; BICAR-ICU Study Group. Sodium bicarbonate therapy for patients with severe metabolic acidaemia in the intensive care unit (BICAR-ICU): a multicentre, open-label, randomised controlled, phase 3 trial. Lancet. 2018;392(10141):31-40.
16. Marschner IC. Confidence distributions for treatment effects in clinical trials: posteriors without prior. Stat Med. 2024;43(6):1271-89.
17. Serpa Neto A, Walker HG, Brown A, Udy A, Bailey M, Marschner I. Beyond neutral trials in critical care: embracing statistical alternatives. Am J Respir Crit Care Med. 2026;212(3):420-7.
18. Cook D, Lauzier F, Rocha MG, Sayles MJ, Finfer S. Serious adverse events in academic critical care research. CMAJ. 2008;178(9):1181-4.
19. Jung B, Jabaudon M, De Jong A, Bitker L, Audard J, Klouche K, et al.; BICARICU-2 Study Group. Sodium bicarbonate for severe metabolic acidemia and acute kidney injury: the BICARICU-2 randomized clinical trial. JAMA. 2025;334(22):2000-10.
20. Neto AS, Fujii T, Moore J, Young PJ, Peake S, Bailey M, et al. Clinical outcomes of Indigenous Australians and New Zealand Māori with metabolic acidosis and acidaemia. Crit Care Resusc. 2023;24(1):14-9.
